# Plasma nucleosome profiling reports on tumor burden and molecular subtypes in small cell lung cancer

**DOI:** 10.1172/JCI202151

**Published:** 2026-10-01

**Authors:** Gavriel Fialkoff, Nobuyuki Takahashi, Israa Sharkia, Jenia Gutin, Nadav Hermoni, Michael Nirula, Rajesh Kumar, Lorinc Pongor, Samantha Nichols, Linda Sciuto, Kanak Parmar, Parth Desai, Priya Suresh, Melissa Abel, Rajaa El Meskini, Myriam Maoz, Yakir Rottenberg, Shoshan Nevo, Hovav Nechushtan, Tamar Peretz, Diana Roame, Ayala Hubert, Jonathan E. Cohen, Azzam Salah, Mark Temper, Albert Grinshpun, Zoe Weaver-Ohler, Arun Rajan, William Douglas Figg, Aviad Zick, Ronen Sadeh, Nir Friedman, Anish Thomas

**Affiliations:** 1 School of Computer Science and Engineering, Hebrew University of Jerusalem, Jerusalem, Israel.; 2 Lautenberg Center for Immunology and Cancer, Institute for Medical Research Israel-Canada, The Hebrew University-Hadassah Medical School, Jerusalem, Israel.; 3 Developmental Therapeutics Branch, Center for Cancer Research, National Cancer Institute (NCI), NIH, Bethesda, Maryland, USA.; 4 Department of Medical Oncology, National Cancer Center Hospital East, Kashiwa, Japan.; 5 Center for Advanced Preclinical Research, Frederick National Laboratory for Cancer Research, NCI, NIH, Frederick, Maryland, USA.; 6 Sharett Institute of Oncology, Hadassah-Hebrew University Medical Center, Jerusalem, Israel.; 7 Genitourinary Malignancies Branch, Center for Cancer Research, and; 8 Thoracic and GI Malignancies Branch, Center for Cancer Research, NCI, NIH, Bethesda, Maryland, USA.; 9 Hebrew University Center for Computational Medicine, The Hebrew University-Hadassah Medical School, Jerusalem, Israel.; 10 Robert H. Lurie Comprehensive Cancer Center and Division of Hematology and Oncology, Department of Medicine, Northwestern University Feinberg School of Medicine, Chicago, Illinois, USA.

**Keywords:** Oncology, Pulmonology, Biomarkers, Cancer, Epigenetics

## Abstract

**BACKGROUND:**

Small-cell lung cancer (SCLC) is an aggressive malignancy with a poor prognosis and marked transcriptional heterogeneity that may drive distinct therapeutic vulnerabilities. Clinical translation of molecular subtyping has been limited by restricted access to tumor biopsies, particularly at relapse.

**METHODS:**

We applied chromatin immunoprecipitation of cell-free nucleosomes carrying active histone modifications followed by sequencing (cfChIP-seq) to 441 plasma samples from individuals with advanced SCLC, other neuroendocrine carcinomas, or non-SCLC cancers, as well as from healthy controls. Plasma cfChIP-seq profiles were integrated with matched tumor transcriptomes from 73 samples, including 41 time-matched pairs.

**RESULTS:**

cfChIP-seq captured the epigenetic and transcriptional landscape of tumor-derived cell-free DNA (cfDNA), including SCLC tissue- and cell-of-origin signatures. A quantitative cfChIP-seq–derived SCLC score tracked radiographic tumor burden and was associated with prognosis. Signals at promoters of lineage-defining transcription factor genes, including *ASCL1*, *NEUROD1*, *POU2F3*, and *ATOH1*, correlated strongly with matched tumor RNA expression and supported noninvasive inference of SCLC transcriptional subtypes directly from plasma.

**CONCLUSION:**

Plasma cfChIP-seq provides a practical liquid biopsy platform for real-time assessment of tumor burden, tumor state, and molecular subtype in SCLC. These findings support further development of cfChIP-seq for precision monitoring and subtype-informed therapeutic stratification in SCLC.

**TRIAL REGISTRATION:**

ClinicalTrials.gov NCT02484404, NCT02487095, NCT02769962, NCT03554473, NCT03896503, and NCT02146170.

**FUNDING:**

Center for Cancer Research; Intramural Program of the NCI (ZIA BC 011793); European Research Council (ERC) (Adg no. 101019560 “cfChIP”).

## Abstract 2

<strong data-end=”325” data-start=”172”>A blood test using chromatin profiling can identify small cell lung cancer subtypes, track tumor burden, and reveal tumor heterogeneity in real time.</strong>

## Introduction

Small-cell lung cancer (SCLC) is a neuroendocrine (NE) lung cancer that is highly aggressive and has a dismal prognosis, accounting for approximately 15% of all lung cancers (1, 2). SCLC is among the solid tumors that sheds the largest amount of cell-free DNA (cfDNA) (3, 4). Prior studies have identified cfDNA mutations in more than 80% of patients with SCLC (5–14), but targetable, recurrent mutations in known oncogenes, such as those in lung adenocarcinoma, are rare in SCLC (15, 16). Recurrent mutations also do not demonstrate consistent co-occurrence or mutual exclusivity and thus do not define SCLC subtypes.

SCLC tumors instead exhibit high expression of neuronal and NE transcription factors and MYC paralogs that drive broad proliferative programs (15, 17, 18). Importantly, SCLC subtypes driven by distinct transcription factors have unique therapeutic vulnerabilities (19–24). However, identification of SCLC transcriptomic subtypes and their application in the context of subtype-specific therapies has proven challenging due to limited access to tumor specimens. The majority of patients with SCLC do not undergo surgical resection because their disease is detected after it has spread beyond the primary site (25). Moreover, patients with relapsed disease generally deteriorate quickly, and recurrence suspected on imaging is typically followed by immediate treatment without biopsies. Highlighting this challenge, SCLC is not represented in any of the large sequencing initiatives, such as The Cancer Genome Atlas (TCGA) and the Pan-cancer Analysis of Whole Genomes (26).

Identifying tumor-specific alterations in cfDNA presents a powerful opportunity for early cancer detection and monitoring, with the potential to reduce cancer morbidity and mortality (27, 28). Most of the current clinical applications of cfDNA are centered around interrogating the mutational landscape and, as such, are of limited utility in defining transcriptomic subtypes. We reported chromatin immunoprecipitation and sequencing of cell-free nucleosomes from human plasma (cfChIP-seq) to infer transcriptional programs by genome-wide mapping of plasma cell–free nucleosomes carrying specific histone modifications (29). Specifically, tri-methylation of histone 3 lysine 4 (H3K4me3) is a well-characterized histone modification marking transcription start sites (TSSs) of genes that are poised or actively transcribed and predictive of gene expression (29–32). Other groups have similarly explored the potential of chromatin and epigenetic features in cfDNA to reconstruct tumor gene regulation and expression states (33, 34), highlighting a growing interest in extending liquid biopsy beyond mutational profiling.

We hypothesized that plasma histone modifications may noninvasively track tumor gene expression, including lineage-defining transcription factors, enabling precision therapy for SCLC. To test this, we applied cfChIP-seq on 441 plasma samples from self-reported healthy individuals (*n* = 33), patients with non–small-cell lung cancer (NSCLC) (*n* = 14), colorectal cancer (CRC) (*n* = 40), NE carcinomas (NECs) (*n* = 47), or SCLC (*n* = 94), who had 195 plasma samples collected at multiple time points during their treatment (Figure 1). Plasma-derived gene expression was compared with matched tumor RNA-seq data (*n* = 54) (Figure 2A and Supplemental Tables 1 and 2). Our analyses revealed a strong correspondence between plasma cfChIP-seq profiles and tumor burden. By focusing on genes defining SCLC subtypes, we demonstrate that plasma cfChIP-seq enabled accurate molecular subtyping, underscoring its potential as a practical biomarker for real-time tumor profiling and precision therapeutic guidance in SCLC. Importantly, our goal is not to replace tissue biopsy–based pathology when adequate tumor material is available, but rather to provide a complementary liquid biopsy approach in clinical scenarios in which tissue is limited, unsafe to obtain, or potentially nonrepresentative due to spatial or temporal tumor heterogeneity.

## Results

### SCLCs have distinct cfChIP-seq signals that track tumor burden and prognosis.

Plasma samples were collected and processed, and H3K4me3 ChIP-seq (29) was performed directly on approximately 1 mL plasma (see Methods) with a mean of 3 million unique reads sequenced per sample (Supplemental Table 3). Library complexity and saturation analysis demonstrated that this depth captured a substantial proportion of the available library, with additional sequencing primarily yielding redundant PCR duplicates (Supplemental Figure 1A). The number of normalized reads mapping to its respective TSS region(s) was computed for every gene, resulting in gene counts analogous to transcription counts in the RNA-seq data. When we compared the gene counts in SCLC plasma samples with those in healthy reference samples (see Methods), we found substantially elevated counts in hundreds to thousands of genes (Figure 2B and Supplemental Figure 1B). To directly assess technical reproducibility within this study, we performed independent cfChIP-seq library preparations and sequencing runs on replicate plasma aliquots from a validation subset of patients (*n* = 11). Tissue signature scores showed high concordance across replicates (median Pearson *r* = 0.99), indicating strong technical robustness of the assay (Supplemental Figure 2).

cfDNA of patients with cancer consists of DNA fragments originating from tumor cells and DNA released by normal cells, primarily from the hematopoietic lineage (29, 35). The tumor-derived fraction of cfDNA can vary substantially, depending on several variables, including tumor burden and growth activity (36). To quantify this variability, we developed an SCLC score that reflects the proportion of tumor-derived cfDNA. The score was generated using a linear regression model that matches the observed gene count profile in a sample to a weighted mixture of reference cfChIP-seq profiles. The reference dataset consisted of plasma samples from healthy individuals and a representative SCLC archetype derived from the 10% of SCLC samples (*n* = 20) with the highest number of significantly elevated gene coverage (Supplemental Figure 1B and Methods).

The SCLC score ranged from 0 (indicating a “healthy-like” profile) to 1 (“SCLC-like”). In SCLC patient samples collected before treatment, the median SCLC score was 0.5, which decreased to 0.11 after treatment, suggesting a reduction in tumor-derived cfDNA due to therapy. In contrast, plasma from healthy individuals and patients with NSCLC displayed absent or very low SCLC scores (median of 0 in healthy and NSCLC, 0.06 in CRC; ANOVA *P* < 10^–15^. Figure 2C). This indicated that the SCLC score reflects tumor-type–specific chromatin programs rather than shared NE gene expression alone.

To ensure that the observed cfChIP-seq differences were driven by tumor biology rather than patient heterogeneity or technical artifacts, we performed a variance driver analysis. Principal component analysis (PCA) across all approximately 25,000 RefSeq genes showed that the first principal component (PC1) accounted for approximately 30% of total variance and was nearly perfectly correlated with the SCLC score (*r* = 0.98, *P* < 10^–15^), identifying tumor burden as the primary driver of the observed signals (Figure 2D). Crucially, clinical variables (age, sex) and technical variables (ChIP batch) showed no significant correlation with PC1 or PC2 (*P >* 0.05). Furthermore, in a multivariate linear model (PC1 ~ SCLC score + age + sex + race + batch), the SCLC score remained the sole dominant predictor of PC1 (*t* = 85, *P* < 10^–16^).

Notably, cfChIP-seq SCLC scores were significantly correlated with multiple other measures of tumor fraction, including genome-wide cfDNA fragmentation–based tumor fraction estimates (37), circulating tumor cell (CTC) counts, total cfDNA concentrations (see Methods; Pearson *r* = 0.78, 0.43, and 0.61; *P* < 2 × 10^–4^, 0.01, and 0.03, respectively), CT scan–based volumetric tumor assessments, and standardized unidimensional tumor measurements (38) (Pearson *r* = 0.58 and 0.61; *P* < 0.007 and <3 × 10^–4^, respectively Figure 2E and Supplemental Figure 1, D and E). Furthermore, cfChIP-seq SCLC scores tracked the radiographic tumor burden through the treatment time course (Figure 2F) and predicted the treatment response and overall survival (Figure 2G and Supplemental Figure 1, F and G).

Taken together, these results demonstrate the potential of cfChIP-seq to noninvasively detect SCLC and quantify the SCLC tumor fraction from plasma. Further investigations are warranted to explore whether this approach could be adapted for lung cancer screening, which may ultimately contribute to improved clinical outcomes.

### cfChIP-seq recovers SCLC tissue and cellular origins.

We next investigated whether plasma cfChIP-seq signals could provide insights into the epigenetic state of tissue and cells of origin of SCLC tumors. Among the more than 3,500 genes with significantly elevated coverage in SCLC plasma (Figure 2B and Methods), many were unique to SCLC. Interestingly, these signals also appeared in plasma from patients with NECs — a histologically related but anatomically diverse group of tumors, highlighting shared transcriptional programs despite distinct tissue origins (39) (Figure 3, A and B).

These SCLC-signature genes showed a remarkable enrichment for genes expressed in SCLC cell lines (40) and pulmonary NE cells (41), with significant overlaps of 272 of 465 and 53 of 92 genes (*P* < 7.8 × 10^–97^ and < 3.5 × 10^–15^, respectively) (42, 43). Specifically, SCLC plasma exhibited elevated counts of canonical SCLC genes such as *DLL3*, *INSM1*, *CHGA*, and *CRMP1*, with significantly higher levels than those observed in healthy samples (Figure 3, C and D).

To characterize the tissue-specific contributions to cfDNA, we established a set of tissue-specific genomic loci using ChIP-seq reference data (44, 45) (see Methods). As expected, signals in plasma from healthy individuals predominantly reflected hematopoietic cells — neutrophils, megakaryocytes, and monocytes (29, 35). In contrast, SCLC plasma exhibited additional signatures indicative of lung, brain, and B cell origins, suggesting contributions from cells of pulmonary, neuronal, and lymphocyte lineages (Figure 3E and Supplemental Figure 3A). These tissue-specific signals positively correlated with the SCLC score, reinforcing their relevance (Supplemental Figure 3B).

To further confirm the cellular origin, we applied lung cell–type–specific signatures derived from single-cell RNA-seq (scRNA-seq) (46). Across the approximately 50 cell types examined, spanning lung epithelial, endothelial, stromal, and immune cells, we observed a strong enrichment of NE cell signatures in SCLC plasma compared with healthy control plasma (Figure 3F). This finding is especially striking, since NE cells constitute only 0.13% of a healthy lung tissue (46). Furthermore, the NE cell signature was markedly elevated in SCLC plasma relative to ChIP-seq results from a bulk sample of normal lung tissue (Figure 3G). Signals from other lung cells, including ciliated cells and alveolar epithelial type 1 cells, were also higher in SCLC plasma compared with healthy control samples (Figure 3F and Supplemental Figure 3, C and D), hinting at the possibility of SCLCs arising additionally from non-NE cells of origin as described previously (47), or indicating injury to the specific cells.

Together, these findings demonstrate that cfChIP-seq can capture the unique epigenetic states of tissues and cells of origin associated with SCLC tumors.

### Plasma cfChIP-seq predicts tumor gene expression of SCLC lineage–defining transcription factors.

Transcriptional signatures of SCLC heterogeneity converge on 2 major cell states, namely NE and non-NE (48). The SCLC cell states are further characterized by the expression of key lineage-defining transcription factors, ASCL1 and NEUROD1, defining NE cell states, and POU2F3, defining non-NE cell states. A fourth subgroup has been characterized by expression of YAP1 (49, 50) or low expression of all 3 transcription factors, accompanied by an inflammatory gene signature (21).

To systematically examine the relationship between the circulating chromatin state and tumor expression of lineage-defining transcription factors, we collected paired cfChIP-seq profiles and RNA-seq data from the same patients. Ideally, we would want time-matched samples, in which both report on the same tumor. However, because of clinical constraints, some comparisons involved RNA-seq from an earlier biopsy and cfChIP-seq at the time of disease recurrence (Supplemental Table 1). To address this, we performed analyses on both time-matched samples (*n* = 41; smaller numbers but better correspondence) and the complete set of samples (*n* = 73; larger numbers but potential for weaker correspondence). Additionally, since the tumor’s contribution to cfDNA may have been low in some cases, we focused on high SCLC score samples, while also considering all available samples.

Most tumors in our cohort exhibited high expression of the NE lineage–defining genes *ASCL1* and *NEUROD1*, with coexpression of both in some cases (Figure 4A). *POU2F3*, *YAP1*, and a newly described subtype marker *ATOH1* (1, 19, 51) were expressed less frequently. To further evaluate transcriptional subtype heterogeneity, we examined the codetection of subtype-defining transcription factors within individual samples using matched tumor RNA-seq and plasma cfChIP-seq. Consistent with dominant subtype assignments, most samples showed enrichment for a single major transcriptional program. However, a subset of cases showed evidence of more than 1 subtype-associated signal, most commonly involving codetection of *ASCL1* and *NEUROD1*. This pattern was observed in both tumor RNA-seq and plasma cfChIP-seq and, in some cases, was more apparent in plasma, consistent with the possibility that cfChIP-seq may capture signals from multiple tumor deposits not fully represented by a single biopsy (Figure 4A and Supplemental Figure 4A).

Given the concordance between chromatin state and gene expression (52–54), we hypothesized that expression of a transcription factor in the tumor would produce a detectable cfChIP-seq signal in plasma, provided that nontumor tissues do not contribute substantially to the same locus. Indeed, cfChIP-seq counts for *ASCL1*, *NEUROD1*, and *POU2F3* were significantly elevated in subsets of SCLC plasma samples, while remaining undetectable in all healthy controls. However, *YAP1* counts were similar among healthy and SCLC plasma samples (Figure 4B and Supplemental Figure 4A), likely due to *YAP1* activity in normal tissue contributing to cfDNA, as suggested by H3K4me3 marks on *YAP1* promoters in normal tissue (44).

To evaluate whether these cfChIP-seq signals reflect tumor gene expression in individual patients, we assessed their correlation with RNA-seq data from tumor samples from individuals with high SCLC scores (*n* = 36). We observed a strong correlation between cfChIP-seq and tumor RNA-seq for *ASCL1*, *NEUROD1*, and *POU2F3* (Pearson *r* = 0.72, 0.72, and 0.86; *P* < 8.2 × 10^–7^,6.2 × 10^–7^, and 1.9× 10^–11,^ respectively), all of which were absent from healthy control cfDNA. A similarly high positive correlation was observed for *ATOH1* (Pearson *r* = 0.82; *P* < 1.3 × 10^–9^). This correlation was observed in both the SCLC and NEC samples (Figure 4C).

Notably, while the TSS of *POU2F3* and *ATOH1* were marked by H3K4me3 in many of the SCLC samples, only in a subset of them did the signals span beyond the TSS region to a wider region of approximately 10 kb, suggesting engagement in cell-type–specific regulatory programs (55). We found that these additional regions correlated best with gene expression in the tumor (Supplemental Figure 4, B and C).

Together, these findings established that plasma cfChIP-seq robustly captured lineage-defining transcription factor expression and subtype heterogeneity in SCLC tumors.

### Subtyping of SCLC from plasma cfChIP-seq.

Next, we sought to explore the possibility of subtyping SCLC tumors directly from plasma using cfChIP-seq. Although SCLC subtyping is often viewed as a discrete classification, we observed cases in which tumors exhibited high levels of 2 or more lineage-defining transcription factors simultaneously (Figure 4B). This aligns with data from recent studies (56). Therefore, we decided to predict the activity of each factor independently, examining 4 distinct classifications for each sample.

We evaluated whether cfChIP-seq signals at individual transcription factor promoter regions could classify subtype identity. Specifically, we used the measured cfChIP-seq level of each transcription factor as a direct proxy for its RNA expression level, without any additional classifier training. By measuring cross-modal concordance between the plasma chromatin and the tumor transcriptome, this approach remained inherently resistant to overfitting and bypassed the need for independent “train test” validation sets. The resulting receiver operating characteristic (ROC) curves demonstrated strong predictive performance for *ASCL1*, *NEUROD1*, *POU2F3*, and *ATOH1*, with high AUC values, even in samples with SCLC scores as low as 0.1 (Figure 5, A and B).

Beyond lineage-defining transcription factors, genome-wide analysis across approximately 25,000 RefSeq promoters demonstrated significant concordance between plasma cfChIP-seq promoter signals and matched tumor RNA-seq profiles, with the strongest agreement observed in high-tumor-fraction samples and tumor-enriched genes, indicating that cfChIP-seq captured a broad tumor-aligned transcriptional program rather than isolated marker loci (Supplemental Figure 5, A and B).

Importantly, cfChIP-seq levels of these transcription factors displayed bimodal behavior as a function of SCLC score (Figure 5A). In negative samples, in which the factor was not expressed, cfChIP-seq signals remained consistently low across SCLC scores. In positive samples, cfChIP-seq levels increased linearly with the SCLC score, suggesting that the signal originated from tumor cells.

Taken together, these findings indicate that cfChIP-seq enabled noninvasive assessment of SCLC transcriptional states and supported molecular subtype inference from plasma. While larger prospective studies and assay standardization are needed, our results establish both the technical feasibility and clinical potential of plasma-based chromatin profiling for disease monitoring and subtype-informed therapeutic decision making.

## Discussion

SCLC is a highly aggressive and genomically unstable malignancy for which molecularly guided treatment remains elusive. This is due, in part, to the near-universal lack of tumor tissue, especially at relapse, which has impeded efforts to define biologically distinct subtypes and identify actionable therapeutic targets. Here, we show that plasma cfChIP-seq, a method that captures active chromatin marks on circulating nucleosomes, overcame this barrier by providing a noninvasive readout of the tumor chromatin state. From as little as 1 mL plasma, cfChIP-seq recovered quantitative SCLC cell–free chromatin signature and lineage-defining transcription factor activity that correlated with matched tumor RNA. These capabilities establish cfChIP-seq as a practical platform for molecular subtyping and disease monitoring in SCLC, with implications for precision therapy.

Our findings demonstrate that SCLC exhibited a distinct, detectable cell-free chromatin signature that distinguished it from other cancers and healthy individuals. This signature was detectable even when tumor-derived cfDNA was low, such as during the treatment response, and correlated strongly with both the clinical tumor burden and the patient’s prognosis.

Importantly, cfChIP-seq profiles captured the activity of key SCLC transcriptional drivers, including ASCL1, NEUROD1, and POU2F3, which define the major SCLC subtypes and underlie divergent therapeutic responses. These results lay the groundwork for noninvasive transcriptional subtyping in clinical settings, potentially enabling more tailored treatments than the current uniform approach. Because longitudinal subtype shifts may reflect both tumor fraction dynamics and true lineage plasticity, plasma-based subtype changes should be interpreted in the context of clinical and molecular covariates and warrant prospective validation with matched tissue when feasible. While prospective studies in larger, treatment-naive and longitudinally sampled cohorts are needed, these findings highlight the feasibility of cfChIP-seq for stratifying SCLC patients on the basis of tumor-intrinsic biology and tracking the evolution of the chromatin landscape throughout the clinical course.

Although SCLC subtyping is often treated as a discrete classification, our data support a more nuanced model in which a subset of tumors shows evidence of more than 1 subtype-associated transcriptional program. Codetection was most commonly observed for *ASCL1* and *NEUROD1*, consistent with prior reports of subtype plasticity and mixed NE states in SCLC. Detection of multiple subtype-associated signals in plasma cfChIP-seq may reflect intratumoral heterogeneity within individual lesions and/or intermetastatic heterogeneity across disease sites. Because plasma cfChIP-seq measures chromatin states from circulating nucleosomes shed into the blood, it may integrate signals from multiple tumor deposits, whereas tumor RNA-seq generally reflects a single sampled biopsy site. These findings suggest that plasma cfChIP-seq could provide a complementary approach for assessing subtype admixture and tumor heterogeneity in patients with SCLC.

Our findings contribute to the broader evaluation of cfDNA-based approaches for tumor characterization. While methylation profiling has enabled subtype classification in SCLC (57, 58), it captures stable, indirect markers of gene regulation and often requires classifier training, limiting its interpretability and adaptability. In contrast, cfChIP-seq directly detects active histone marks linked to gene transcription, offering mechanistic insight into the tumor state. Notably, the classification reported here did not involve classifier training or the selection of informative features. Exploiting the direct connections between gene activity and chromatin state at the gene level, we were able to use cfChIP-seq signal levels at a transcription factor’s promoter to predict its activity in the tumor.

Fragmentation-based methods, such as nucleosome positioning (59) or fragmentomics (59, 60), have also shown promise for detecting chromatin features in cfDNA, but their utility appears more limited. We have found that fragmentomics could infer the expression of select transcription factors such as *NEUROD1* and *REST* but lacked the broader resolution needed for comprehensive subtyping (59). cfChIP-seq overcomes this by capturing a wide spectrum of transcriptional regulators, enabling more robust and clinically actionable molecular profiling directly from plasma.

Overall, our study bridges the gap between molecular studies and the treatment of patients with SCLC, an exceptionally lethal malignancy, which to date is treated as a homogenous disease with identical treatments for all patients. Moreover, this work suggests the applicability of cfChIP-seq to a wider context to profile and subtype tumors in a way that can be transformative for patient care across multiple cancer types.

## Methods

### Sex as a biological variable

Both male and female participants were included in this study (see Table 1 for sex distribution by cohort). Sex was self-reported by the participants. To evaluate sex as a potential confounder, we performed variance-driver analysis and multivariable linear regression. These analyses demonstrated that sex was not a significant driver of variance in our primary biological outcomes (*P >* 0.05), and tumor-derived signals remained the dominant predictor regardless of sex. Consequently, our findings are representative of and applicable to both sexes.

### Patients

We undertook an observational study using plasma collected from patients with SCLC who received care at the NCI, NIH. Patients were enrolled in therapeutic clinical trials (ClinicalTrials.gov NCT02484404; NCI protocol no. 15-C-0145; ClinicalTrials.gov NCT02487095; NCI protocol no. 15-C-0150; ClinicalTrials.gov NCT02769962; NCI protocol no. 16-C-0107; ClinicalTrials.gov NCT03554473; NCI protocol no. 18-C-0110; and ClinicalTrials.gov NCT03896503; NCI protocol no. 20-C-0009). We also collected samples from patients with small-cell cancer who were enrolled in the NCI thoracic malignancies natural history protocol (ClinicalTrials.gov NCT02146170; NCI protocol no. 14-C-0105). See Supplemental Table 1 for information on each patient. If tumor samples were available, we also sequenced their RNA at matched or different time points at which the plasma was collected. Samples from patients with CRC were collected at Hadassah Medical Center in Jerusalem. Patients were offered participation in protocol HMO-0346-12, titled “Characterization of Genetic Material, including Total Genome/Exome Sequencing, from Tumor Cells as a Predictive and Prognostic Measure, and a Tool to Follow Up Treatment in Cancer Patients.” For patients who provided consent, a blood sample was obtained for analysis.

### Demographic reporting

Demographic and technical variables, including age, sex, race and ethnicity, and processing batch, are detailed in Table 1. Race and ethnicity were recorded on the basis of clinical records. For the healthy and CRC patient cohorts, specific subethnicities were not collected; these participants represent a general Middle Eastern population (Israel-based). For the SCLC and NEC cohorts, inclusive demographic data are reported; where specific categories were unavailable or participants chose not to disclose, they were categorized as “other (self-identified)” to ensure transparent and inclusive reporting.

Smoking history was documented for all patient and control cohorts when available (Table 1). For cohorts or individuals for whom smoking history was not systematically collected or was partially incomplete, the status is explicitly designated as “NA” or “incomplete” within the clinical characteristics matrices.

### Tumor RNA-seq

Formalin-fixed, paraffin-embedded (FFPE) tumor tissue samples or frozen tumor samples in selected samples were prepared for RNA-seq. RNA enrichment was performed using the TruSeq RNA Exome Library Prep according to the manufacturer’s instructions (Illumina). Paired-end sequencing (2 × 75 bp) was performed on an Illumina NextSeq 500 instrument. RNA was extracted from FFPE tumor cores using RNeasy FFPE kits according to the manufacturer’s protocol (QIAGEN). RNA-seq libraries were generated using TruSeq RNA Access Library Prep Kits (TruSeq RNA Exome kits; Illumina) and sequenced on NextSeq 500 sequencers using a 75 bp paired-end sequencing method (Illumina). Reads were aligned to the human genome (hg19) using STAR (2.7.10a) (61). For transcriptomics analyses, raw RNA-seq count data were normalized for inter-gene/inter-sample comparisons using trimmed mean of M-values normalized counts per million (TMM-CPM), as implemented in the edgeR R/Bioconductor package (62).

### Fragmentomic-based ctDNA estimation

Estimation of ctDNA fraction in the plasma samples was performed using the DNA evaluation of fragments for early interception method as previously described (60).

### CTCs

CTCs were detected from 10 mL peripheral blood drawn into EDTA tubes. Epithelial cell adhesion molecule–positive (EpCAM^+^) CTCs were isolated using magnetic pre-enrichment and quantified using multiparameter flow cytometry. CTCs were identified as viable, nucleated, EpCAM^+^ cells that did not express the common leukocyte antigen CD45, as described previously (22).

### Radiological volumetric segmentation

We performed volumetric segmentation, a 3D assessment of CT, as previously described (63). This method may more accurately predict clinical outcomes than conventional evaluation by Response Evaluation Criteria in Solid Tumors (RECIST). Briefly, experienced radiologists reviewed the CT sequences to determine the best ones for segmentation using the lesion management application in PACS (Vue PACS, version 12.0, Carestream Health). Lesion size was also assessed using RECIST, version 1.1.

### Plasma cfChIP-seq

#### Sample collection.

Plasma for cfDNA was collected in EDTA tubes and centrifuged at 4°C at 1,500*g* for 10 minutes. Plasma was carefully transferred into Eppendorf tubes, centrifuged a second time at 4°C at 10,000*g* for 10 minutes, and transferred into standard cryovials.

#### Bead preparation.

ChIP was performed using the following antibodies: anti-H3K4me3 (07-473, MilliporeSigma), anti-H3K4me3 (C15410003-50, Diagenode), and a proprietary anti-H3K4me3 antibody provided by Senseera Ltd. A total of 50 μg antibody was conjugated to 5 mg epoxy M270 Dynabeads (Invitrogen, Thermo Fisher Scientific) according to the manufacturer’s instructions. The antibody-bead complexes were kept at 4ºC in PBS, 0.02% azide solution.

#### Immunoprecipitation, NGS library preparation, and sequencing.

Immunoprecipitation, library preparation, and sequencing were performed by Senseera Ltd. using previously reported protocols (29), with certain modifications that increase capture and the signal-to-background ratio. Briefly, the designated ChIP antibodies were covalently immobilized to paramagnetic beads and incubated with plasma. Barcoded sequencing adaptors were ligated to chromatin fragments, and DNA was isolated and next-generation sequenced.

To assess technical reproducibility, independent library preparations and sequencing were performed for a subset of the cohort (*n* = 11). For these samples, 2 separate aliquots from the same plasma collection were processed in parallel across independent batches. Reproducibility was quantified using Pearson correlation coefficients of the resulting promoter-level signals.

#### Sequencing analysis.

Reads were aligned to the human genome (hg19) using bowtie2 (2.3.4.3) with “no-mixed” and “no-discordant” flags. We discarded fragment reads with low alignment scores (–q 2) and duplicate fragments. See Supplemental Table 3 for read numbers, alignment statistics, and the number of unique fragments for each sample.

Preprocessing of sequencing data was performed as previously described (29). Briefly, the human genome was segmented into windows representing the TSS, flanking to the TSS, and the background (rest of the windows). The fragments covering each of these regions were quantified and used for further analysis. Nonspecific fragments were estimated per sample and extracted, resulting in the specific signal in every window. Counts were normalized and scaled to 1 million reads in healthy reference accounting for sequencing depth differences.

### Statistics

#### Genes elevated in SCLC versus healthy plasma.

A reference of healthy plasma gene baseline and statistical test to evaluate whether a gene was significantly elevated compared with the baseline were computed as previously described (29).

#### SCLC score.

To estimate SCLC score reflecting the tumor-related fraction in samples, we performed a leave-one-out non-negative least squares test using the “nnls” R package (1.4).

Denoting by the number of gene promoters and by the number of samples in the reference cohort, a matrix *X*_GX(*S*+1)_ was composed that contained the counts per promoter in the *S* healthy samples, with the addition of a “SCLC prototype” composed on the mean promoter counts of the 10% SCLC samples, with most genes having significantly elevated coverage compared to healthy plasma (*n* = 15). For every vector *Y*_1XG_ of counts per promoter in the SCLC samples, we estimated the non-negative coefficient *β* by computing *argmax_β_* ||*Xβ* – *Y*||^2^ subject to *β* ≥0. A similar process is computed for healthy samples with the exception that for the *i_th_* sample, we used the matrix *X_–i_* where the column containing that sample was eliminated. The estimated β was normalized to 1 by computing
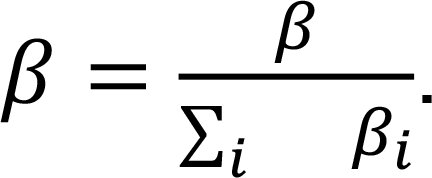


The tumor-related fraction was defined as the value of *β_SCLC_* **—** the fraction assigned to the SCLC prototype. The healthy fraction of the *i_th_* sample was defined as Σ_i≠SCLC_ *β_i_* — the sum of fractions assigned to all other healthy components.

An alternative approach was tested using a reference atlas of roadmap tissue data. In this approach, the healthy fraction is set to be the sum of fractions assigned to common tissues observed typically in healthy samples (neutrophils, monocytes, megakaryocytes) and the tumor-related fraction is defined as 1 — the tumor-related fraction. The 2 approaches resulted in very similar estimations for the vast majority of samples (data not shown).

We compute the principal PCA of all gene counts in all SCLC and healthy samples as implemented in the R “prcomp” function. Scree plot was generated using the “fviz_eig” function of the R “factoextra” package, version 1.0.7.

#### Tissue signatures.

The genomic regions selected for tissue-specific markers were as described previously (29). cfDNA from patients with SCLC consisted of tumor-related cfDNA above the hematopoiesis-derived cfDNA observed in healthy individuals. Estimation of the absolute contribution of tissues to the cfDNA pool was calculated by multiplying the normalized signal per signature and the estimated number of reads in the sequencing library size (29), which approximates the cfDNA concentration.

#### Lung single-cell signatures.

To define pulmonary cell-type–specific signatures, we made use of published pulmonary scRNA-seq data (46) using cluster-specific marker gene sets defined by the authors. To increase the specificity of the cell-type signature in the cfDNA context, we include only genes that met the following criteria: (a) The percentage of cells outside the cluster that express the genes was less than 0.1; (b) the average log fold-change expression in the cluster compared with other clusters was greater than 2; (c) the adjusted *P* value of the gene was less than 0.1; (d) the mean gene promoter read counts in the cfDNA in healthy reference was less than 3. The last criterion aimed to remove from the signature any genes that were not lung specific but appeared in circulation as part of normal cell turnover. A summary of the genes included in every lung cell-type signature can be found in Supplemental Table 5.

#### Subtyping of plasma samples.

On the basis of the RNA-seq distribution, we defined the following CPM cutoffs: *ASCL1* = 10, *NEUROD1* = 60, *POU2F3* = 40, and *ATOH1* = 4. Plasma samples with matching tumors were annotated as TF^+^ for each of the 4 TFs.

### Study approval

This observational study used plasma and tumor samples collected from patients with SCLC who received care at the NCI, NIH. Patients were enrolled in therapeutic clinical trials, including ClinicalTrials.gov NCT02484404/NCI protocol 15-C-0145; NCT02487095/NCI protocol 15-C-0150; NCT02769962/NCI protocol 16-C-0107; NCT03554473/NCI protocol 18-C-0110; and NCT03896503/NCI protocol 20-C-0009. Additional samples were collected from patients enrolled in the NCI thoracic malignancies natural history protocol, ClinicalTrials.gov NCT02146170/NCI protocol 14-C-0105. The studies were approved by the human subjects committee/IRB at the NCI, NIH. All participants provided written informed consent for the collection and sequencing of plasma, tumor, and matched normal samples, as applicable.

### Data availability

The source data and individual data points underlying all reported means and graphical representations presented in the main manuscript figures and supplemental materials are provided in the Supporting Data Values Excel file. This file is available alongside the online version of this article as part of the supplemental material, with separate, clearly labeled tabs corresponding to each applicable figure panel.

Plasma cfChIP-seq normalized promoter coverage and tumor RNA-seq CPM values are available in the Zenodo repository (https://zenodo.org/records/13766909). Genomic visualization tracks (BigWig files) of the cfChIP-seq data can be viewed interactively via a dedicated UCSC Genome Browser session (https://genome.ucsc.edu/s/nirfriedman/SCLC%20cfChIP%2Dseq). Alternatively, users can manually load the tracks by pasting the Track Hub configuration file URL (https://files.cs.huji.ac.il/nirf/track_hubs/SCLC-cfChIP/hub.txt) into the ‘Connected Hubs’ interface of the UCSC Genome Browser.

Plasma cfChIP-seq data from SCLC, NEC, and NSCLC samples have been deposited in the European Genome-phenome Archive (EGA). Overarching metadata are available under study ID EGAS50000001916 for SCLC, NEC, and NSCLC samples, and under study ID EGAS00001004913 for CRC and healthy control samples. Primary sequencing files are available under dataset ID EGAD50000002783 for SCLC, NEC, and NSCLC samples, and dataset ID EGAD00001006811 for CRC and healthy control samples. Tumor RNA-seq data have been deposited in the Database of Genotypes and Phenotypes (dbGaP) under accession number phs003628.v1.p1.

#### Code availability.

All script files used for analyzing data for this manuscript are available on https://github.com/Nir-Friedman-Lab/SCLC_cfChIPseq; commit ID fffef8cf.

## Author contributions

GF, NT, RS, NF, and AT conceived and designed the study. GF, IS, JG, NH, RS, and NF developed and performed cfChIP-seq analyses and computational methods. NT, MN, RK, LP, SN, LS, KP, PD, PS, MA, AR, WDF, and AT contributed to clinical sample acquisition, clinical data curation, patient annotation, and interpretation of clinical correlates. RE and ZWO contributed to preclinical research resources and sample-related support. MM, YR, NS, HN, TP, AH, JEC, AS, MT, AG, and AZ contributed patient samples, clinical annotations, and interpretation of the Hadassah cohort. DR contributed clinical and correlative study support. GF, NT, RS, NF, and AT interpreted the data and wrote the manuscript with input from all authors. NF and AT supervised the study. All authors reviewed and approved the final manuscript.

### Role of funding source

The funding sources had no role in the design or conduct of the study; collection, management, analysis, or interpretation of the data; preparation, review, or approval of the manuscript; or the decision to submit the manuscript for publication. The contributions of the NIH author(s) are considered Works of the United States Government. The findings and conclusions presented in this paper are those of the author(s) and do not necessarily reflect the views of the NIH or the US Department of Health and Human Services.

## Conflict of interest

AT received funding from EMD Serono, AstraZeneca, Gilead Sciences, and ProLynx during the conduct of the study.

## Funding support

This work is the result of NIH funding, in whole or in part, and is subject to the NIH Public Access Policy. Through acceptance of this federal funding, the NIH has been given a right to make the work publicly available in PubMed Central.

- NIH Intramural Research Program (NCI ZIA BC 011793 to AT).
- European Research Council (ERC Adg no. 101019560 “cfChIP,” to NF).
- Israel Science Foundation (ISF) (personal grant no. 2279/22, to AZ).
- ISF Israel Precision Medicine Partnership (IPMP) (grant no. 3099/22, to AZ).
- TRANSCAN-3 Project “EpiNanSarc,” co-funded by the Israel Ministry of Health and European Union Horizon 2020 (to AZ).

## Supplementary Material

Supplemental data

ICMJE disclosure forms

Supplemental tables 1-5

Supporting data values

## Figures and Tables

**Figure 1:**
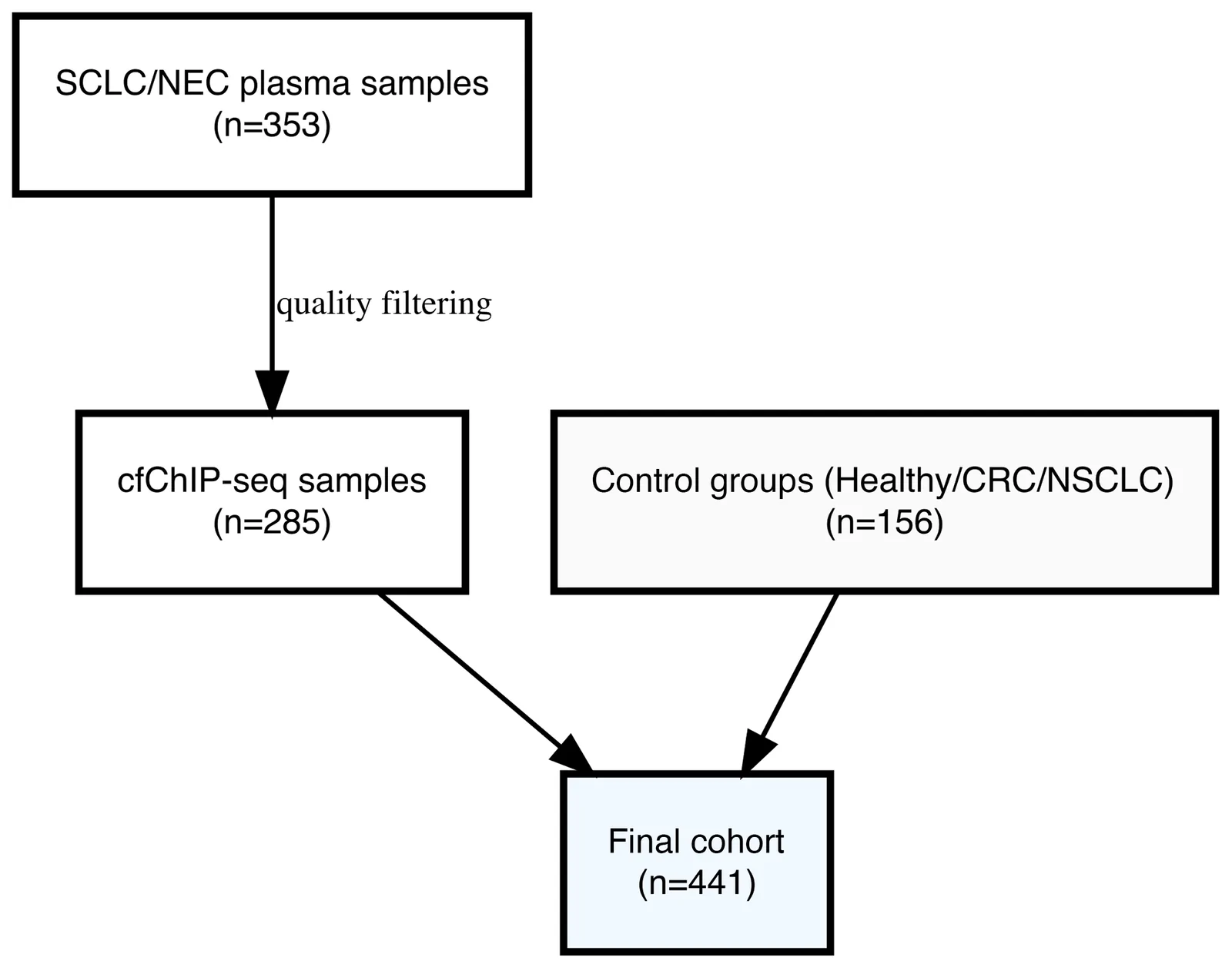
Flow diagram of patient cohorts and sample selection. Visual overview of sample inclusion criteria and quality filtering pipelines. The chart traces the consolidation of the initial SCLC/NEC plasma pool (*n* = 353) down to a filtered cfChIP-seq subset (*n* = 285), which was merged with diverse control groups (*n* = 156) to yield the final analysis cohort (*n* = 441).

**Figure 2:**
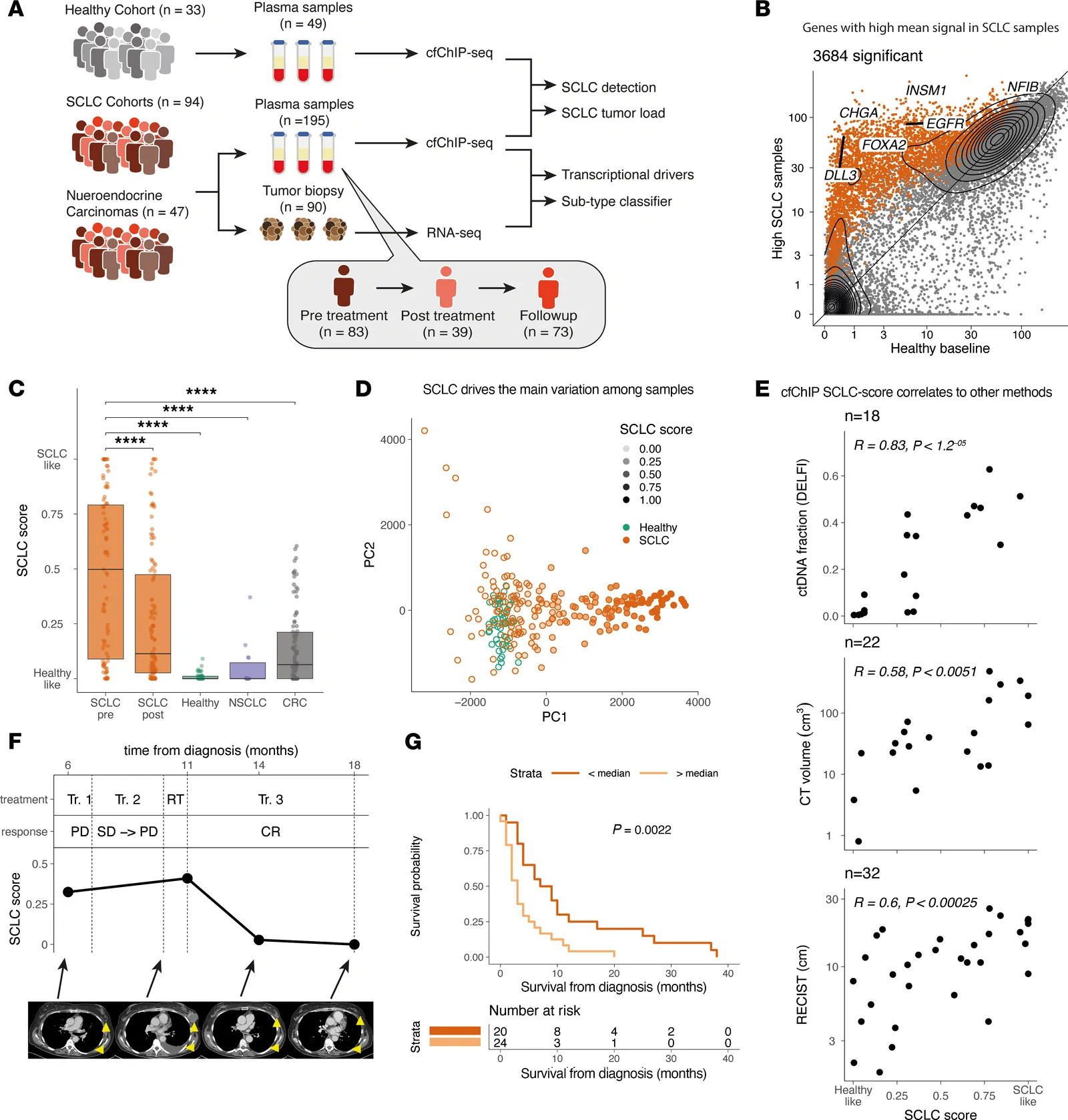
SCLC plasma samples exhibit distinct cfChIP-seq signals that correlate with tumor burden and survival. (**A**) Study outline. Plasma and tumor samples were collected from a cohort of healthy individuals and patients with SCLC at various time points during treatment. cfChIP-seq was performed on plasma samples and RNA-seq on tumor biopsy samples. Plasma cfChIP-seq–based gene expression profiles were benchmarked against tumor RNA-seq, and key SCLC transcription regulators were examined. (**B**) Genes with significantly high coverage in selected SCLC (*n* = 20) samples (see Methods) compared with healthy baseline values. For each gene, we compared mean normalized coverage in the SCLC samples (*y* axis) with a healthy cohort reference (*x* axis). A total of 3,197 genes with significantly higher coverage (*q* < 0.001) in at least 6 SCLC samples are marked (see Supplemental Table 4). Significance was determined as previously described (29). (**C**) Median SCLC score for different cohorts calculated by linear regression (see Methods). Each dot is a sample, and box plots summarize each group’s SCLC score distribution. The horizontal line represents the median, the box bounds represent the 25th and 75th percentiles, and the whiskers extend to the largest and smallest values within 1.5 times of the IQR. Pre/post refers to the timing of the collection relative to treatment. ****P < 0.0001, by Kruskal-Wallis test. (**D**) Principal components 1 and 2 of SCLC (orange) and healthy (green) samples. Transparency indicates SCLC score as in **C**. PCA was done using all Refseq genes (~25,000). This representative plot represents a single analysis of the cohort. (**E**) Correlation of the SCLC score with other plasma and imaging-based measures of tumor burden. Pearson correlation coefficients and *P* values were determined using the ggpubr stat_cor function. DELFI, DNA evaluation of fragments for early interception. (**F**) Changes in the SCLC score and radiological tumor burden in a patient with SCLC over the treatment time course (SCLC0191). Tr. 1, durvalumab and olaparib; Tr. 2, topotecan and M6620; RT, radiation therapy; Tr. 3, investigational therapy; CR, complete response; SD, stable disease; PD, progressive disease. (**G**) Kaplan-Meier survival plot for patients with an SCLC score above (red; median survival: 3 months) or below (blue; median survival: 8.5 months) the median. The survival curve was generated using pretreatment samples. *P* value was calculated by log-rank test. This survival analysis was performed once on the patient cohort.

**Figure 3:**
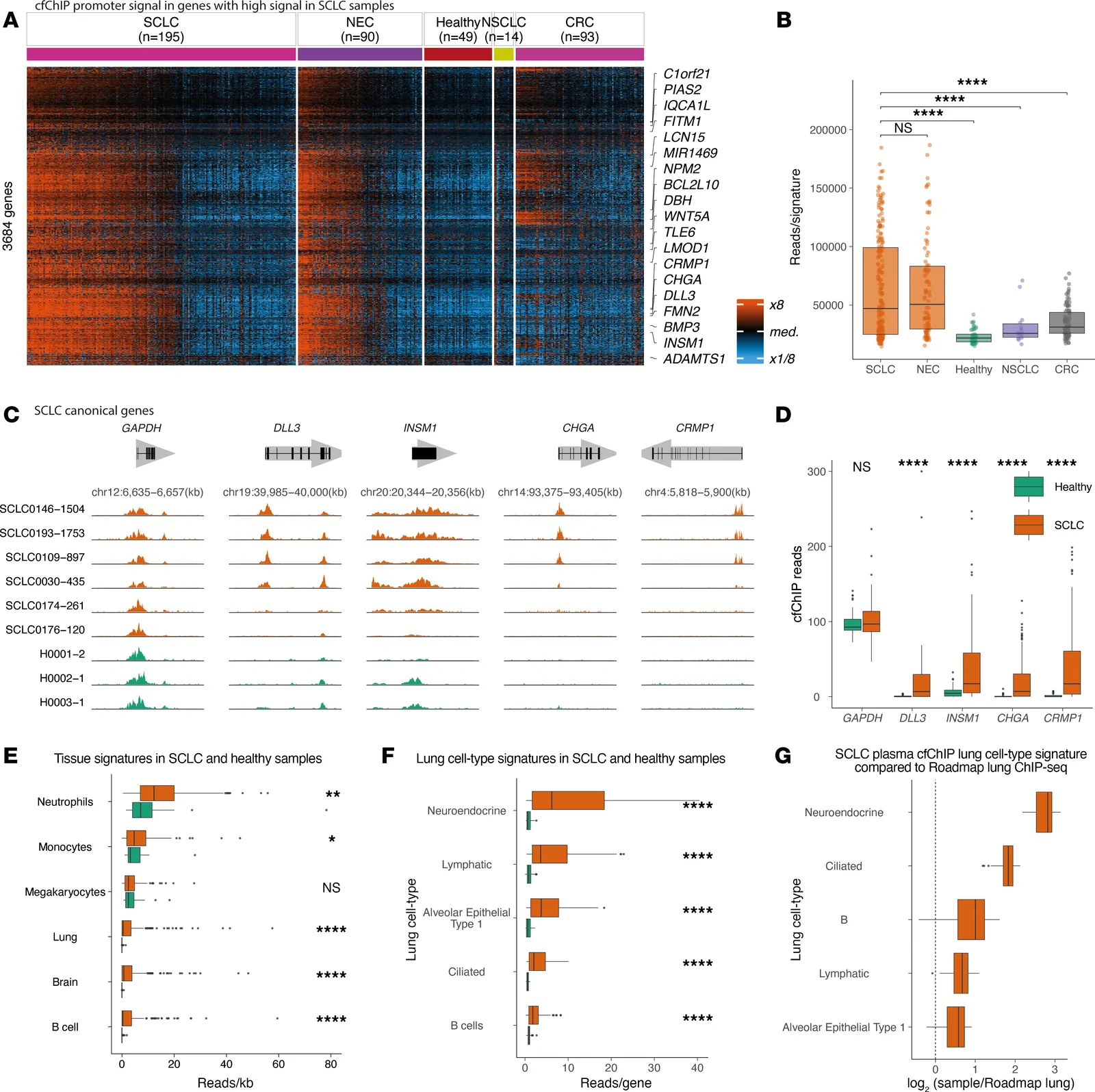
cfChIP-seq recovers SCLC tissue and cellular origins. (**A**) Hierarchical clustering of SCLC signature genes (rows) across the study cohorts (columns). Samples within each cohort are ordered by the SCLC score. Normalized coverage on the gene promoter was log transformed [log_2_(1+coverage)] and adjusted to the zero mean for each gene across the samples. (**B**) Median and distribution of coverage on the genes shown in **A** across the study cohorts. Box plots are as defined in Figure 1. ****P < 0.0001, by Wilcoxon rank-sum test. (**C**) Genome browser view of cfChIP-seq signal in canonical SCLC genes (*DLL3*, *INSM1*, *CHGA*, *CRMP1*), with *GAPDH* as the control. Orange and green tracks represent SCLC and healthy samples, respectively. (**D**) Median and distribution of the cumulative cfChIP-seq coverage over the SCLC signature genes. Box plots are as defined in Figure 1. ****P < 0.0001, by Wilcoxon rank-sum test. (**E**) Cell- and tissue-of-origin signatures in healthy and SCLC samples. The *x* axis indicates the absolute contribution of the signature (normalized reads/kb corrected by the estimated cfDNA concentration; see Methods). Box plots are as defined in Figure 1. *P <0.05, **P < 0.01, and ****P < 0.0001, by Wilcoxon rank-sum test. (**F**) scRNA-seq–derived lung cell-type signatures in healthy and SCLC samples. The *x* axis indicates the sum of normalized reads in the marker genes of every tissue. Horizontal lines represent the median, box bounds represent the 25th and 75th percentiles, and whiskers extend to 1.5 times the IQR. ****P < 0.0001, by Wilcoxon rank-sum test. (**G**) Ratio for signal in the cell types shown in **F** of SCLC samples with an SCLC score of greater than 0.5 compared with Roadmap lung ChIP-seq. Box plots are as defined in Figure 1.

**Figure 4:**
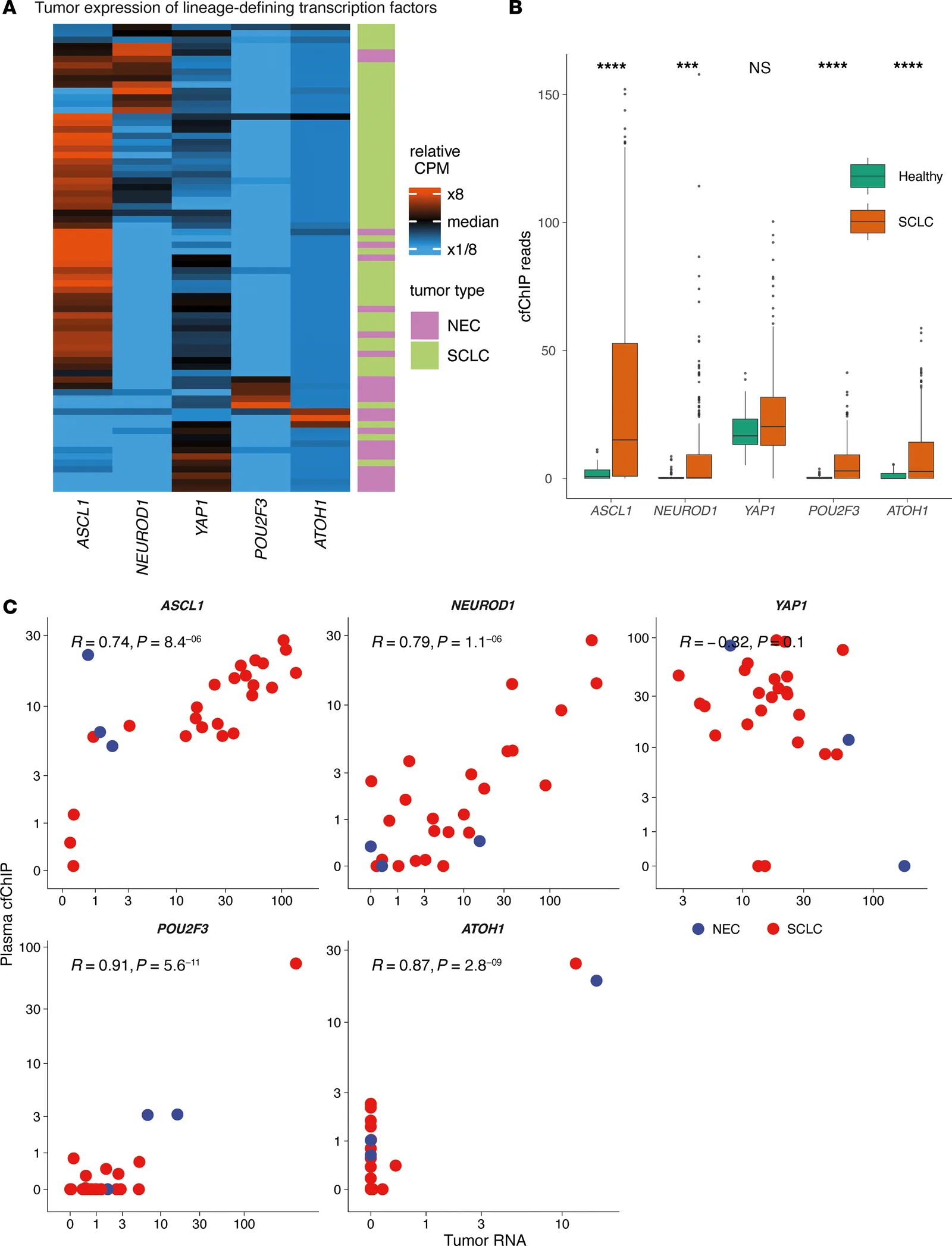
cfChIP-seq displays differential expression of SCLC transcription drivers. (**A**) Heatmap displaying the normalized expression levels of the canonical SCLC transcription factor drivers across tumor RNA-seq samples. To clearly distinguish active from inactive transcriptional states, expression values for each individual transcription factor were normalized relative to an empirically defined threshold established at the inflection point separating the on/off bimodal distribution of the cohort samples. Consistent with distinct lineage programs, expression patterns of these transcription drivers demonstrate a generally mutually exclusive distribution across individual tumors. (**B**) Median and distribution of SCLC and healthy cfChIP-seq plasma sample coverage on the genes shown in **A**. Box plots are as defined in Figure 1. ****P < 0.0001, by Wilcoxon rank-sum test. (**C**) Correlation of plasma cfChIP-seq coverage and tumor RNA-seq of the genes shown in **C** across time-matched plasma samples with an SCLC score above 0.5 (*n* = 27). Color indicates the tumor type. The correlation coefficient and *P* value were determined by Pearson correlation using the ggpubr stat_cor function.

**Figure 5:**
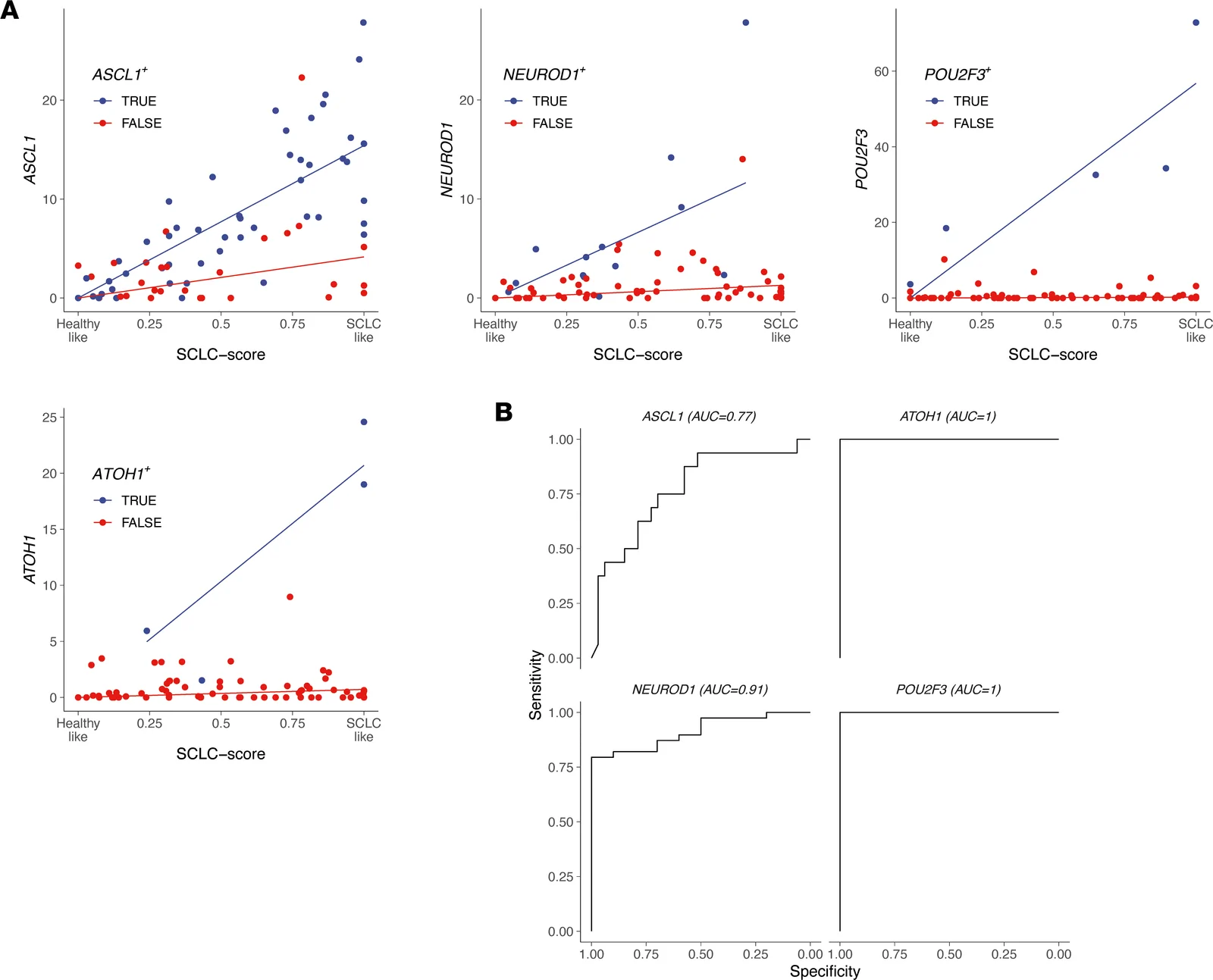
SCLC subtyping using cfChIP-seq signals. (**A**) SCLC score (*x* axis) and cfChIP-seq promoter coverage (*y* axis) of samples that are annotated as positive (blue) and negative (red) to NEUROD1, POU2F3, and ATOH1 based on biopsy RNA-seq. Linear fit shows a positive relation between the promoter coverage and SCLC score across the positive samples while the negative samples remain low and constant (*n* = 73 paired samples). Statistical significance was determined by Pearson correlation. This analysis was conducted once. (**B**) ROC plots summarizing the performance of the single-gene classifiers for plasma samples with SCLC score of greater than 0.1 (*n* = 49).

**Table 1:**
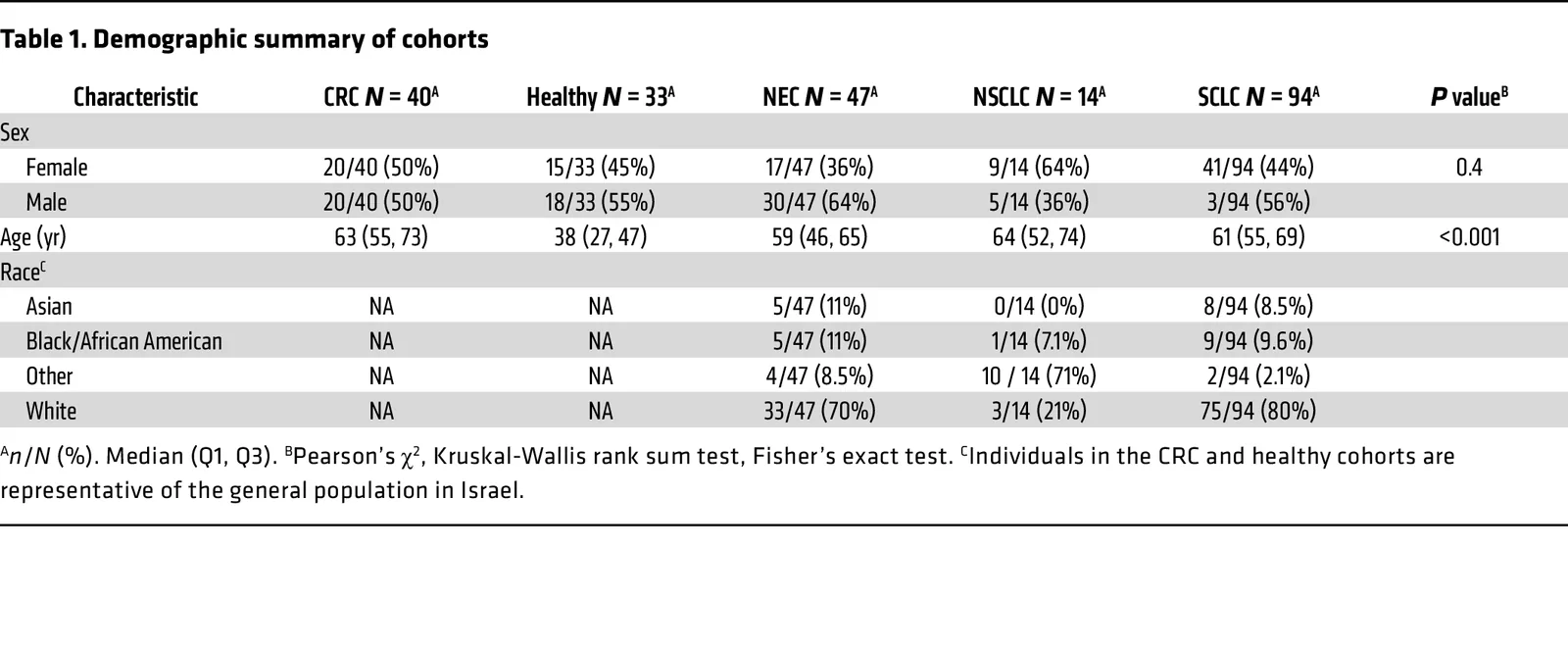
Demographic summary of cohorts

## Version 1 10/01/2026

Electronic publication

## References

[1] Rudin CM (2021). Small-cell lung cancer. Nat Rev Dis Primers.

[2] Thomas A (2025). Advancing therapeutics in small-cell lung cancer. Nat Cancer.

[3] Zill OA (2018). The landscape of actionable genomic alterations in cell-free circulating tumor DNA from 21,807 advanced cancer patients. Clin Cancer Res.

[4] Shi J (2021). Genomic landscape and tumor mutational burden determination of circulating tumor DNA in Over 5,000 Chinese patients with lung cancer. Clin Cancer Res.

[5] Almodovar K (2018). Longitudinal cell-free DNA analysis in patients with small cell lung cancer reveals dynamic insights into treatment efficacy and disease relapse. J Thorac Oncol.

[6] Nong J (2018). Circulating tumor DNA analysis depicts subclonal architecture and genomic evolution of small cell lung cancer. Nat Commun.

[7] Mohan S (2020). Profiling of circulating free DNA using targeted and genome-wide sequencing in patients with SCLC. J Thorac Oncol.

[8] Mondelo-Macía P (2021). Current status and future perspectives of liquid biopsy in small cell lung cancer. Biomedicines.

[9] Fernandez-Cuesta L (2016). Identification of circulating tumor DNA for the early detection of small-cell lung cancer. EBioMedicine.

[10] Devarakonda S (2019). Circulating tumor DNA profiling in small-cell lung cancer identifies potentially targetable alterations. Clin Cancer Res.

[11] Board RE (2008). Isolation and extraction of circulating tumor DNA from patients with small cell lung cancer. Ann N Y Acad Sci.

[12] Du M (2018). Genomic alterations of plasma cell-free DNAs in small cell lung cancer and their clinical relevance. Lung Cancer.

[13] Iams WT (2017). Longitudinal monitoring of circulating tumor DNA and peripheral T cell repertoire in patients with small cell lung cancer. J Clin Oncol.

[14] Hou JM (2012). Clinical significance and molecular characteristics of circulating tumor cells and circulating tumor microemboli in patients with small-cell lung cancer. J Clin Oncol.

[15] George J (2015). Comprehensive genomic profiles of small cell lung cancer. Nature.

[16] Liu Q (2024). Proteogenomic characterization of small cell lung cancer identifies biological insights and subtype-specific therapeutic strategies. Cell.

[17] Kim D-W (2018). Transcriptional deregulation underlying the pathogenesis of small cell lung cancer. Transl Lung Cancer Res.

[18] Pongor LS (2023). Extrachromosomal DNA amplification contributes to small cell lung cancer heterogeneity and is associated with worse outcomes. Cancer Discov.

[19] Rudin CM (2019). Molecular subtypes of small cell lung cancer: a synthesis of human and mouse model data. Nat Rev Cancer.

[20] Roper N (2021). Notch signaling and efficacy of PD-1/PD-L1 blockade in relapsed small cell lung cancer. Nat Commun.

[21] Gay CM (2021). Patterns of transcription factor programs and immune pathway activation define four major subtypes of SCLC with distinct therapeutic vulnerabilities. Cancer Cell.

[22] Thomas A (2021). Therapeutic targeting of ATR yields durable regressions in small cell lung cancers with high replication stress. Cancer Cell.

[23] Lissa D (2022). Heterogeneity of neuroendocrine transcriptional states in metastatic small cell lung cancers and patient-derived models. Nat Commun.

[24] Immune heterogeneity in small-cell lung cancervulnerability to immune checkpoint blockade (2024). Immune heterogeneity in small-cell lung cancer and vulnerability to immune checkpoint blockade. Cancer Cell.

[25] Thomas A (2018). Characteristics and outcomes of small cell lung cancer detected by CT screening. Chest.

[26] Thomas A (2022). Translational research: A patient-centered approach to bridge the valley of death. Cancer Cell.

[27] Corcoran RB (2019). Aberrant RNA Splicing in Cancer. Annu Rev Cancer Biol.

[28] Sun K (2015). Plasma DNA tissue mapping by genome-wide methylation sequencing for noninvasive prenatal, cancer, and transplantation assessments. Proc Natl Acad Sci U S A.

[29] Sadeh R (2021). ChIP-seq of plasma cell-free nucleosomes identifies gene expression programs of the cells of origin. Nat Biotechnol.

[30] Karlić R (2010). Histone modification levels are predictive for gene expression. Proc Natl Acad Sci U S A.

[31] Weiner A (2015). High-resolution chromatin dynamics during a yeast stress response. Mol Cell.

[32] Liu CL (2005). Single-nucleosome mapping of histone modifications in S. cerevisiae. PLoS Biol.

[33] Baca SC (2023). Liquid biopsy epigenomic profiling for cancer subtyping. Nat Med.

[34] Sipola J (2025). Plasma cell-free DNA chromatin immunoprecipitation profiling depicts phenotypic and clinical heterogeneity in advanced prostate cancer. Cancer Res.

[35] Moss J (2018). Comprehensive human cell-type methylation atlas reveals origins of circulating cell-free DNA in health and disease. Nat Commun.

[36] Bettegowda C (2014). Detection of circulating tumor DNA in early- and late-stage human malignancies. Sci Transl Med.

[37] Cristiano S (2019). Genome-wide cell-free DNA fragmentation in patients with cancer. Nature.

[38] Eisenhauer EA (2009). New response evaluation criteria in solid tumours: revised RECIST guideline (version 1.1). Eur J Cancer.

[39] Wang Z (2024). Molecular subtypes of neuroendocrine carcinomas: A cross-tissue classification framework based on five transcriptional regulators. Cancer Cell.

[40] Barretina J (2012). The Cancer Cell Line Encyclopedia enables predictive modelling of anticancer drug sensitivity. Nature.

[41] Cao J (2020). A human cell atlas of fetal gene expression. Science.

[42] Chen EY (2013). Enrichr: interactive and collaborative HTML5 gene list enrichment analysis tool. BMC Bioinformatics.

[43] Kuleshov MV (2016). Enrichr: a comprehensive gene set enrichment analysis web server 2016 update. Nucleic Acids Res.

[44] Roadmap Epigenomics Consortium (2015). Integrative analysis of 111 reference human epigenomes. Nature.

[45] Stunnenberg HG (2016). The international human epigenome consortium: a blueprint for scientific collaboration and discovery. Cell.

[46] Travaglini KJ (2020). A molecular cell atlas of the human lung from single-cell RNA sequencing. Nature.

[47] Ferone G (2020). Cells of origin of lung cancers: lessons from mouse studies. Genes Dev.

[48] Zhang W (2018). Small cell lung cancer tumors and preclinical models display heterogeneity of neuroendocrine phenotypes. Transl Lung Cancer Res.

[49] McColl K (2017). Reciprocal expression of INSM1 and YAP1 defines subgroups in small cell lung cancer. Oncotarget.

[50] Qu S (2022). Molecular subtypes of primary SCLC tumors and their associations with neuroendocrine and therapeutic markers. J Thorac Oncol.

[51] Simpson KL (2020). A biobank of small cell lung cancer CDX models elucidates inter- and intratumoral phenotypic heterogeneity. Nat Cancer.

[52] Heintzman ND (2007). Distinct and predictive chromatin signatures of transcriptional promoters and enhancers in the human genome. Nat Genet.

[53] Heintzman ND (2009). Histone modifications at human enhancers reflect global cell-type-specific gene expression. Nature.

[54] Lara-Astiaso D (2014). Immunogenetics. Chromatin state dynamics during blood formation. Science.

[55] Benayoun BA (2015). H3K4me3 breadth is linked to cell identity and transcriptional consistency. Cell.

[56] Baine MK (2020). SCLC Subtypes Defined by ASCL1, NEUROD1, POU2F3, and YAP1: a comprehensive immunohistochemical and histopathologic characterization. J Thorac Oncol.

[57] Chemi F (2022). cfDNA methylome profiling for detection and subtyping of small cell lung cancers. Nat Cancer.

[58] Heeke S (2024). Tumor- and circulating-free DNA methylation identifies clinically relevant small cell lung cancer subtypes. Cancer Cell.

[59] Takahashi N (2025). Genomic alterations and transcriptional phenotypes in circulating free DNA and matched metastatic tumor. Genome Med.

[60] Skidmore ZL (2023). Dissecting small cell lung cancer subtypes with cell-free DNA fragmentomes. J Clin Oncol.

[61] Dobin A (2013). STAR: ultrafast universal RNA-seq aligner. Bioinformatics.

[62] Robinson MD (2010). edgeR: a Bioconductor package for differential expression analysis of digital gene expression data. Bioinformatics.

[63] Fenerty KE (2016). Predicting clinical outcomes in chordoma patients receiving immunotherapy: a comparison between volumetric segmentation and RECIST. BMC Cancer.

