# Supplementary material for: Plasma nucleosome profiling reports on tumor burden and molecular subtypes in small cell lung cancer
Source: J Clin Invest. 2026 Oct 1;136(19):e202151. doi: 10.1172/JCI202151 (PMC13626826; doi:10.1172/JCI202151)
Supplement: Supplementary file 1 [file jci-136-202151-s210.pdf]

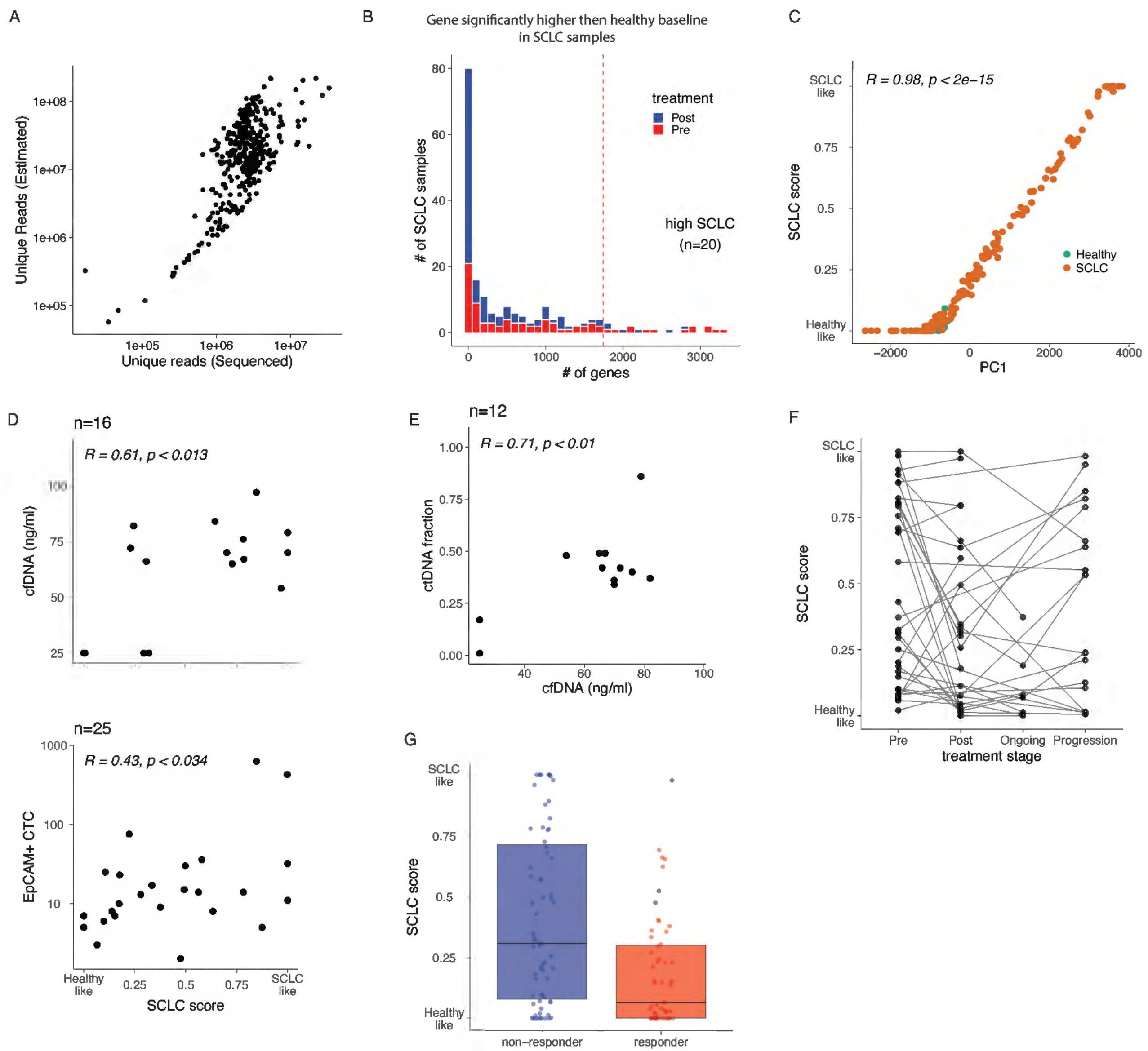

A
Unique Reads (Estimated)
Unique reads (Sequenced)
B
Gene significantly higher then healthy baseline in SCLC samples
# of SCLC samples
# of genes
treatment
Post
Pre
high SCLC (n=20)
C
R = 0.98, p < 2e−15
SCLC score
PC1
Healthy
SCLC
D
n=16
R = 0.61, p < 0.013
cfDNA (ng/ml)
n=25
R = 0.43, p < 0.034
EpCAM+ CTC
SCLC score
E
n=12
R = 0.71, p < 0.01
ctDNA fraction
cfDNA (ng/ml)
F
SCLC score
treatment stage
Pre
Post
Ongoing
Progression
G
SCLC score
non−responder
responder

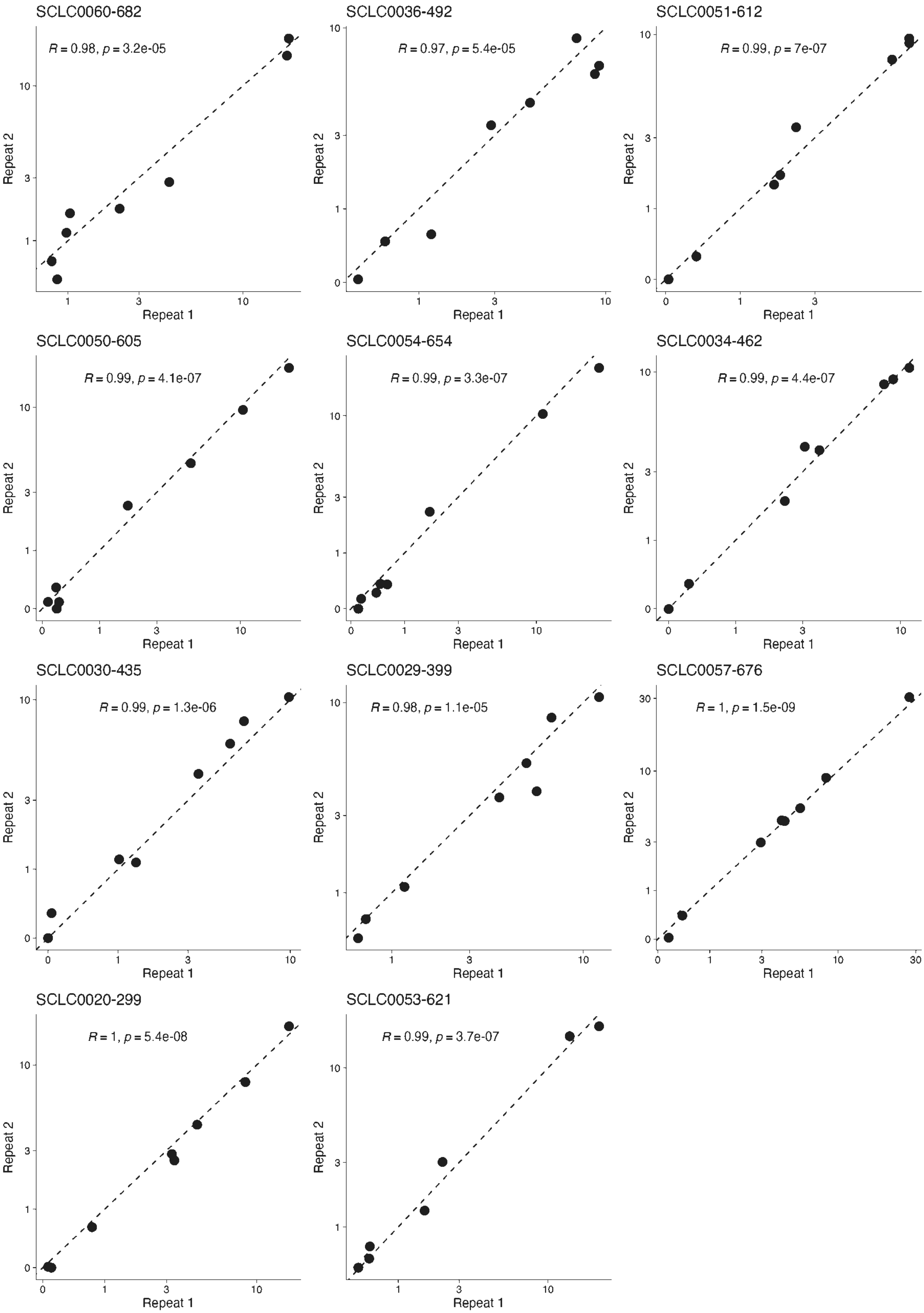
SCLC0060-682
R = 0.98, p = 3.2e-05
SCLC0036-492
R = 0.97, p = 5.4e-05
SCLC0051-612
R = 0.99, p = 7e-07
SCLC0050-605
R = 0.99, p = 4.1e-07
SCLC0054-654
R = 0.99, p = 3.3e-07
SCLC0034-462
R = 0.99, p = 4.4e-07
SCLC0030-435
R = 0.99, p = 1.3e-06
SCLC0029-399
R = 0.98, p = 1.1e-05
SCLC0057-676
R = 1, p = 1.5e-09
SCLC0020-299
R = 1, p = 5.4e-08
SCLC0053-621
R = 0.99, p = 3.7e-07
Repeat 1
Repeat 2

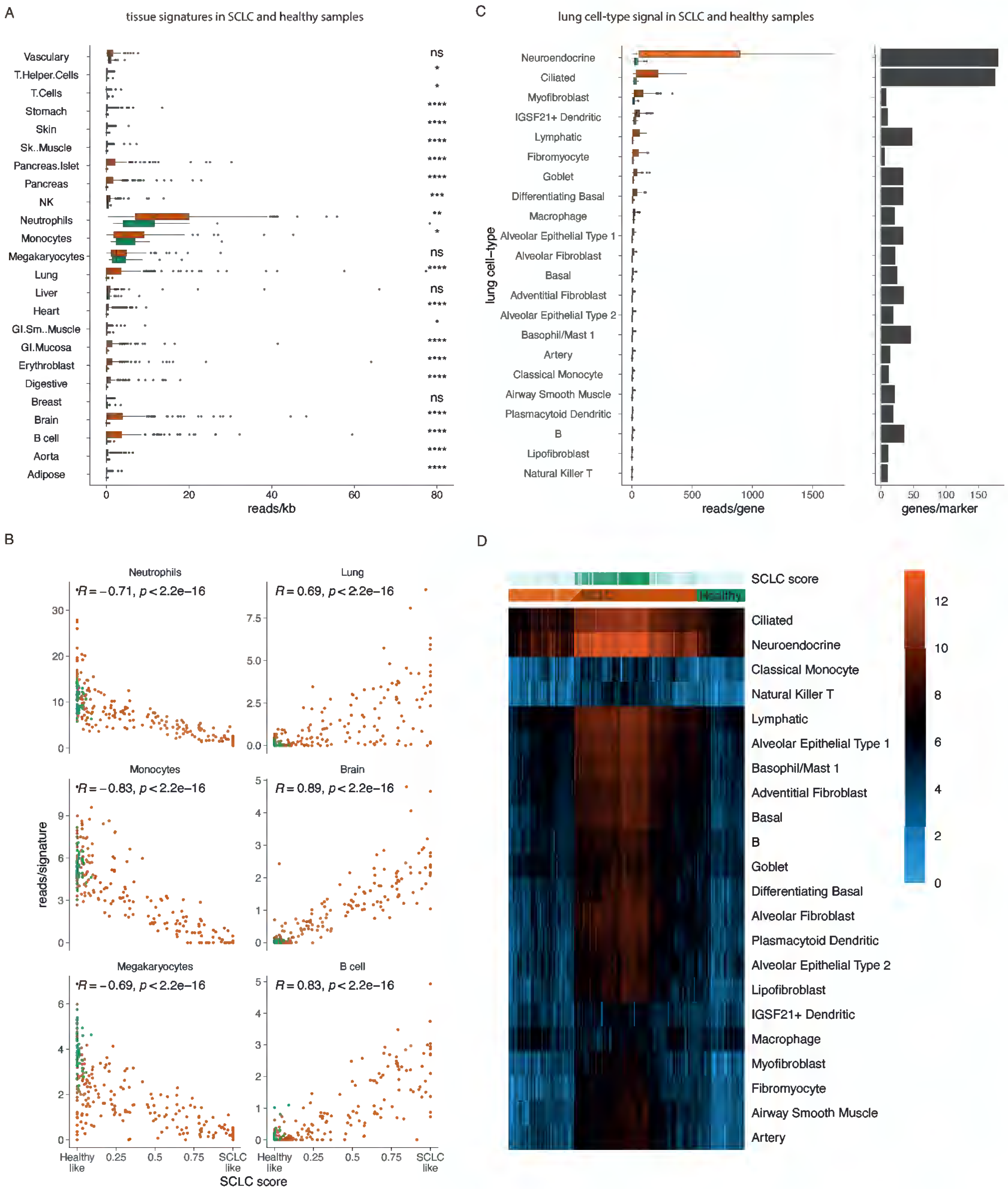
A
tissue signatures in SCLC and healthy samples
Vasculary
T.Helper.Cells
T.Cells
Stomach
Skin
Sk..Muscle
Pancreas.Islet
Pancreas
NK
Neutrophils
Monocytes
Megakaryocytes
Lung
Liver
Heart
GI.Sm..Muscle
GI.Mucosa
Erythroblast
Digestive
Breast
Brain
B cell
Aorta
Adipose
ns
*
*
****
****
****
****
****
***
**
*
ns
****
ns
****
*
****
****
****
ns
****
****
****
****
0
20
40
60
80
reads/kb
B
Neutrophils
R = −0.71, p < 2.2e−16
Lung
R = 0.69, p < 2.2e−16
Monocytes
R = −0.83, p < 2.2e−16
Brain
R = 0.89, p < 2.2e−16
Megakaryocytes
R = −0.69, p < 2.2e−16
B cell
R = 0.83, p < 2.2e−16
reads/signature
Healthy like
0.25
0.5
0.75
SCLC like
SCLC score
C
lung cell-type signal in SCLC and healthy samples
lung cell−type
Neuroendocrine
Ciliated
Myofibroblast
IGSF21+ Dendritic
Lymphatic
Fibromyocyte
Goblet
Differentiating Basal
Macrophage
Alveolar Epithelial Type 1
Alveolar Fibroblast
Basal
Adventitial Fibroblast
Alveolar Epithelial Type 2
Basophil/Mast 1
Artery
Classical Monocyte
Airway Smooth Muscle
Plasmacytoid Dendritic
B
Lipofibroblast
Natural Killer T
0
500
1000
1500
reads/gene
0
50
100
150
genes/marker
D
SCLC score
SCLC
Healthy
Ciliated
Neuroendocrine
Classical Monocyte
Natural Killer T
Lymphatic
Alveolar Epithelial Type 1
Basophil/Mast 1
Adventitial Fibroblast
Basal
B
Goblet
Differentiating Basal
Alveolar Fibroblast
Plasmacytoid Dendritic
Alveolar Epithelial Type 2
Lipofibroblast
IGSF21+ Dendritic
Macrophage
Myofibroblast
Fibromyocyte
Airway Smooth Muscle
Artery
12
10
8
6
4
2
0

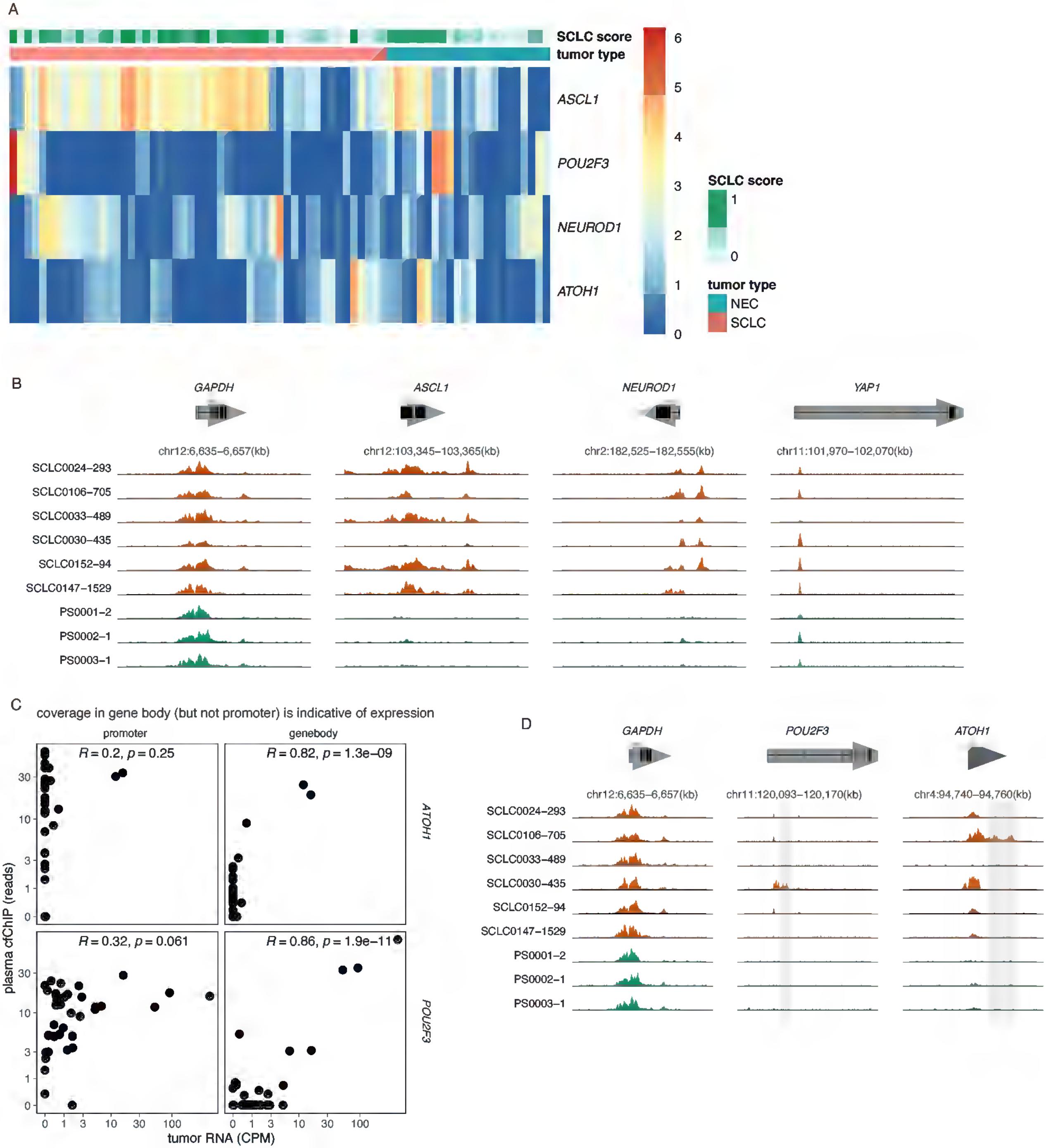
A
SCLC score
tumor type
ASCL1
POU2F3
NEUROD1
ATOH1
6
5
4
3
2
1
0
SCLC score
1
0
tumor type
NEC
SCLC
B
GAPDH
ASCL1
NEUROD1
YAP1
chr12:6,635–6,657(kb)
chr12:103,345–103,365(kb)
chr2:182,525–182,555(kb)
chr11:101,970–102,070(kb)
SCLC0024–293
SCLC0106–705
SCLC0033–489
SCLC0030–435
SCLC0152–94
SCLC0147–1529
PS0001–2
PS0002–1
PS0003–1
C
coverage in gene body (but not promoter) is indicative of expression
promoter
genebody
R = 0.2, p = 0.25
R = 0.82, p = 1.3e–09
R = 0.32, p = 0.061
R = 0.86, p = 1.9e–11
ATOH1
POU2F3
plasma cfChIP (reads)
tumor RNA (CPM)
0
1
3
10
30
100
D
GAPDH
POU2F3
ATOH1
chr12:6,635–6,657(kb)
chr11:120,093–120,170(kb)
chr4:94,740–94,760(kb)
SCLC0024–293
SCLC0106–705
SCLC0033–489
SCLC0030–435
SCLC0152–94
SCLC0147–1529
PS0001–2
PS0002–1
PS0003–1

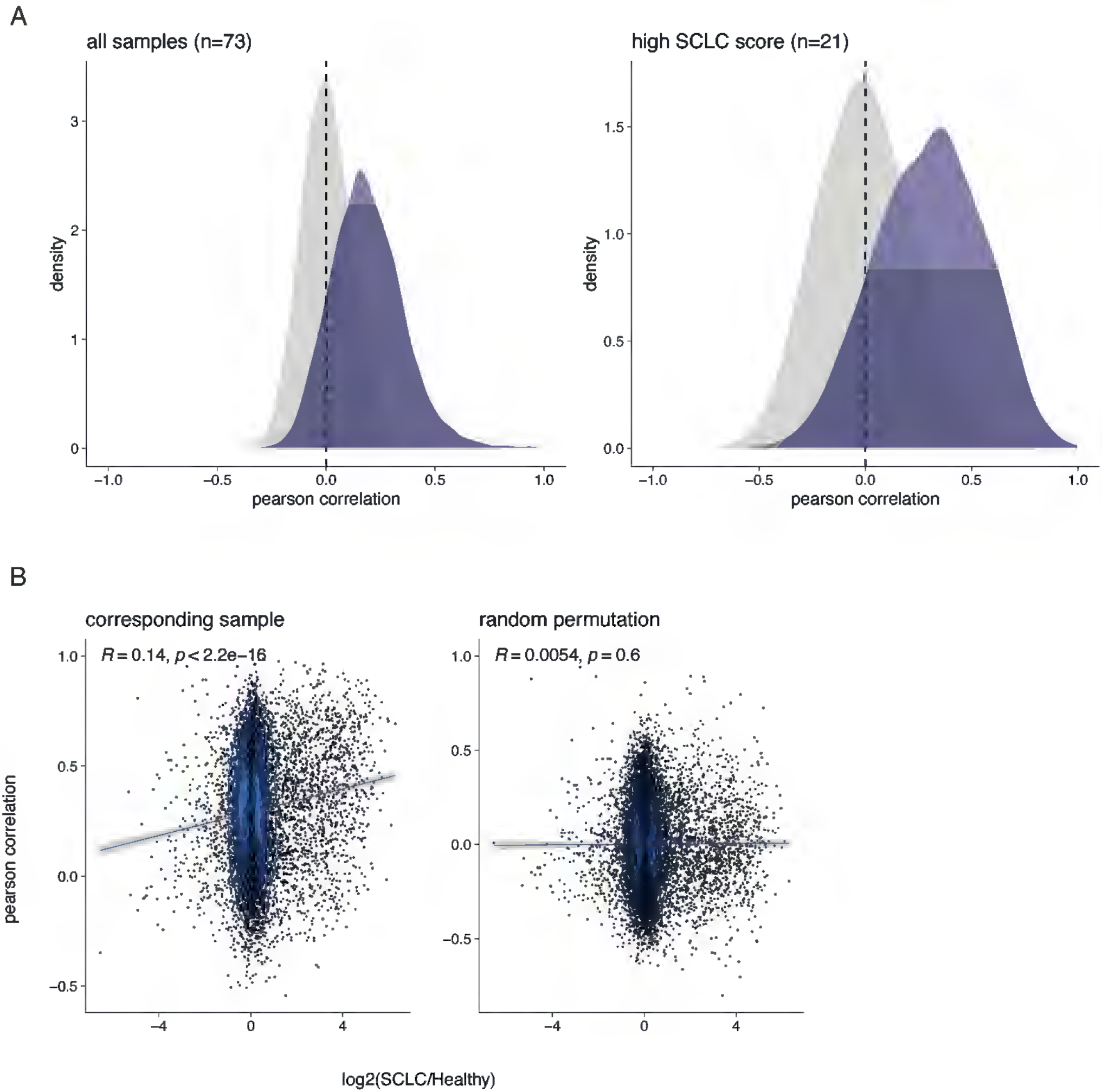
A
all samples (n=73)
high SCLC score (n=21)
density
pearson correlation
B
corresponding sample
random permutation
R = 0.14, p < 2.2e−16
R = 0.0054, p = 0.6
pearson correlation
log2(SCLC/Healthy)

Supplementary Figure Legends

Fig. S1: SCLC-score correlates with tumor load and predicts response to treatment

A. Comparison of sequenced unique reads (x-axis) against the estimated total unique molecules in the library (y-axis); the distribution demonstrates that the majority of the available molecular library has been captured. Analysis was conducted once for the entire cohort.
B. Distribution of the number of genes with significantly higher coverage in SCLC samples compared to healthy baseline (Methods). Dashed line represents the 90th percentile..
C. Correlation of PC1 and SCLC-score (presented in fig 1C-D). High correlation indicates that the main variation in the data is driven by the SCLC contribution to the cfDNA. Pearson correlation and P value were determined by stat_cor.
D. Correlation of SCLC-score and other plasma-based methods for tumor load estimations (cfDNA concentration, circulating tumor cells). Pearson correlation and P value were determined by stat_cor.
E. Correlation of cfDNA concentration and ctDNA fraction. Pearson correlation and P value were determined by stat_cor.
F. Dynamics of SCLC-score during treatments of topotecan and berzosertib (an ATR inhibitor) (ClinicalTrial.gov identifier NCT02487095). Gray lines indicate multiple time points of the same individual.

Median SCLC-score in patients that did or did not respond to various investigational treatments: combination of topotecan and berzosertib (NCT02487095, NCT03896503); olaparib and durvalumab (NCT02484404); nanoparticle camptothecin (CRLX101) and olaparib (NCT 02769962); M7824 (PD-1 inhibitor and TGF-B trapping) and topotecan or temozolomide (NCT03554473). Each dot is a sample, and boxplots summarize distribution of values in each group. Abbreviations: ATR: ataxia telangiectasia and Rad3-related. Boxplots are as defined in Figure 1.

Figure S2: Technical Reproducibility of Tissue-of-Origin Signatures.

Scatter plots correlate normalized tissue-specific signatures across independent replicates for n=11 SCLC and NEC patients. Each replicate represents a discrete library preparation and sequencing run from a single plasma aliquot. Data points indicate normalized contributions of hematopoietic, lung, neuronal, and SCLC-specific lineages. Median Pearson correlation (r = 0.99) across the validation cohort confirms the technical stability of cfChIP-seq and its resistance to batch-level variation during library processing and sequencing. Data are presented as dot plots. Statistical significance and correlation coefficients were determined by Pearson correlation using the ggpubr stat_cor function. Each scatter plot represents the results of two independent technical replicates per patient.

Fig. S3: SCLC tissue and cell of origin

A. Distribution of signal for cell-type signatures (same as fig. 2E). boxplots as defined in Figure 1. P value by Wilcoxon rank-sum test. **** $p < 0.0001$.
B. Correlation of cell-type signature (shown in fig 2E) and SCLC-score. Cell-types observed only in SCLC samples are positively correlated with SCLC-score, while cell-types observed in healthy samples are negatively correlated to SCLC-score. boxplots as defined in Figure 1. P value by Wilcoxon rank-sum test. **** $p < 0.0001$. Pearson correlation coefficients and P values were determined using the stat_cor function.
C. Left: number of marker genes used for every cell-type (Methods; **Supplementary Table 5**). Right: Distribution of signal for lung cell-type marker genes (same as fig. 2F). **** and ***: P < 0.0001 and < 0.001, respectively. boxplots as defined in Figure 1. P value by Wilcoxon rank-sum test.
D. Heatmap showing patterns of signal in lung cell-type markers in the SCLC and healthy samples. Color represents log2(1+cumulative coverage in promoters of marker genes).

Fig. S4: cfChIP signal in SCLC lineage-driving genes

A. Heatmap displaying the promoter enrichment profiles of the 4 major subtype-defining transcription factors which are absent in healthy control plasma. Rows represent individual transcription factors, and columns represent individual plasma cfChIP-seq samples from patients with matched tumor profiling. Color intensity corresponds to the log2(1 + normalized promoter coverage). Top annotation tracking bars indicate the corresponding tumor DNA burden estimate for each sample (SCLC score) and the confirmed clinical histology or tumor type (small-cell lung cancer [SCLC] vs neuroendocrine carcinoma [NEC]). While most profiles demonstrate dominant single-subtype enrichment, a distinct subset of cases displays multi-subtype signal co-detection (predominantly ASCL1 and NEUROD1 dual-positivity), capturing localized or metastatic tumor deposit heterogeneity not fully reflected by single core tissue biopsies.
B. Genome browser view of cfChIP-seq signal in SCLC key transcriptional regulators.

C. Correlation of plasma cfChIP-seq coverage in promoter and gene body and tumor RNA-seq of the genes *POU2F3* and *ATOH1*. Noticeably, in these genes, the correlation to expression is higher in the gene body. Data are presented as dot plots. Pearson correlation coefficients and P values were determined using the stat_cor function.
D. Genome browser view of cfChIP-seq signal in POU2F3 and ATOH1. Orange and green tracks represent SCLC and healthy samples respectively. While a signal in the gene promoter is observed in many samples, only in specific samples there is a significant coverage also in the gene body.

Figure S5: Genome-wide Concordance Between Tumor Transcriptome and Plasma Chromatin

A. Density plots showing the Pearson correlation between tumor RNA-seq and plasma cfChIP-seq across ~25,000 RefSeq genes. The left panel includes the entire cohort (n=73), while the right panel focuses on samples with high tumor fraction (SCLC-score > 0.5; n=21). The observed correlation (blue) is significantly higher than expected by chance, as demonstrated by random sample permutations (gray; $P < 10^{-16}$). The center line of the density plot represents the distribution of Pearson correlation coefficients.
B. Bottom: Correlation between RNA-seq and cfChIP-seq (y-axis) plotted against gene-level SCLC specificity (ratio of coverage in SCLC vs. healthy plasma). The increasing correlation confirms that the alignment between plasma and tissue scales with tumor-specific signal intensity and is most robust for genes uniquely expressed by the tumor. Data is presented as a dot plot. Statistical significance was determined by Pearson correlation using the stat_cor function. This analysis was conducted once.

Supplementary information

- Supplementary Table 1 - Metadata of SCLC patients, blood samples and biopsies used in this study
- Supplementary Table 2 - Sample category (SCLC, NEC, NSCLC, CRC, healthy) for samples used in this study
- Supplementary Table 3 - Sequencing statistics for samples sequenced in this study
- Supplementary Table 4 - SCLC differential genes
- Supplementary Table 5 - Lung cell-type marker genes used in Figure 2F-G and S2C

Data availability

The source data and individual data points underlying all reported means and graphical representations presented in the main manuscript figures and supplementary materials are provided as a single Excel file, titled 'Supporting data values'. This file is available alongside the online version of this article as part of the supplemental material, with separate, clearly labeled tabs corresponding to each applicable figure panel.

Plasma cfChIP-seq normalized promoter coverage and tumor RNA-seq cpm values are available in the Zenodo repository 10.5281/zenodo.13766908. UCSC Track hub with cfChIP tracks is available at https://files.cs.huji.ac.il/nirf/track_hubs/SCLC-cfChIP/hub.txt. See also https://genome.ucsc.edu/s/nirfriedman/SCLC%20cfChIP%2Dseq.

Plasma cfChIP-seq data from SCLC, neuroendocrine carcinoma (NEC), and non-small-cell lung cancer (NSCLC) samples have been deposited in the European Genome-phenome

Archive (EGA). Overarching metadata are available under study ID EGAS50000001916 for SCLC, NEC, and NSCLC samples, and under study ID EGAS00001004913 for colorectal cancer and healthy control samples. Primary sequencing files are available under dataset ID EGAD50000002783 for SCLC, NEC, and NSCLC samples, and dataset ID EGAD00001006811 for colorectal cancer and healthy control samples. Tumor RNA-seq data have been deposited in the database of Genotypes and Phenotypes (dbGaP) under accession number phs003628.v1.p1.

Code availability

All script files used for analyzing data of this manuscript are available on https://github.com/Nir-Friedman-Lab/SCLC_cfChIPseq.
